# Deciphering the Molecular Mechanism Underpinning Phage Arbitrium Communication Systems

**DOI:** 10.1016/j.molcel.2019.01.025

**Published:** 2019-04-04

**Authors:** Francisca Gallego del Sol, José R. Penadés, Alberto Marina

**Affiliations:** 1 Instituto de Biomedicina de Valencia (IBV-CSIC) and CIBER de Enfermedades Raras (CIBERER), 46010 Valencia, Spain; 2 Institute of Infection, Immunity and Inflammation, College of Medical, Veterinary and Life Sciences, University of Glasgow, Glasgow G12 8TA, UK

**Keywords:** arbitrium system, lysis, lysogeny, temperate phage, decision-making, RRNPP, quorum sensing, protein plasticity, phage communication

## Abstract

*Bacillus* phages use a communication system, termed “arbitrium,” to coordinate lysis-lysogeny decisions. Arbitrium communication is mediated by the production and secretion of a hexapeptide (AimP) during lytic cycle. Once internalized, AimP reduces the expression of the negative regulator of lysogeny, AimX, by binding to the transcription factor, AimR, promoting lysogeny. We have elucidated the crystal structures of AimR from the *Bacillus subtilis* SPbeta phage in its apo form, bound to its DNA operator and in complex with AimP. AimR presents intrinsic plasticity, sharing structural features with the RRNPP quorum-sensing family. Remarkably, AimR binds to an unusual operator with a long spacer that interacts nonspecifically with the receptor TPR domain, while the HTH domain canonically recognizes two inverted repeats. AimP stabilizes a compact conformation of AimR that approximates the DNA-recognition helices, preventing AimR binding to the *aimX* promoter region. Our results establish the molecular basis of the arbitrium communication system.

## Highlights

• Crystal structures of AimR in its apo, AimP-, and DNA-bound states were determined
• AimR belongs to the RRNPP family and presents high plasticity for DNA recognition
• AimR operators present unusually long spacers recognized by the AimR TPR domain
• AimP stabilizes a compact conformation of AimR, preventing operator recognition

## Graphical Abstract

**Figure:**
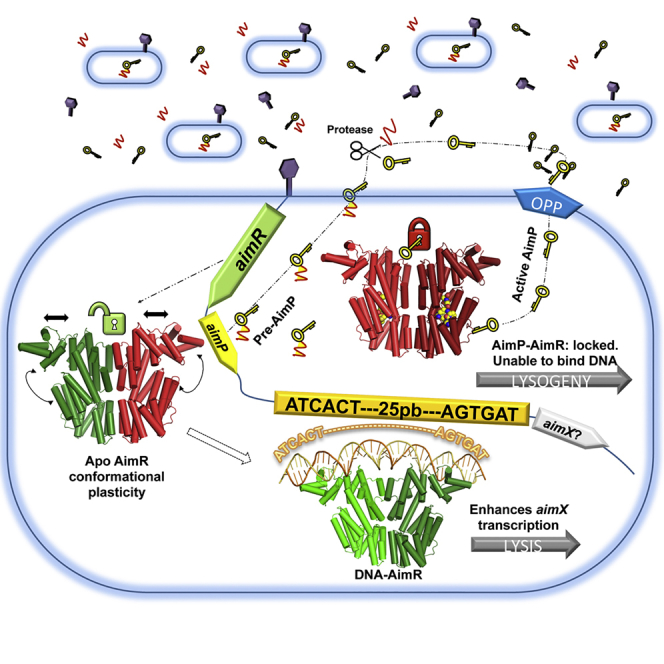

## Teaser

AimR is the sensor receptor of the arbitrium lysis-lysogeny decision system. Through structural and functional analyses, Gallego del Sol et al. reveal that intrinsic plasticity of AimR, which is restricted by the inhibitory AimP peptide, allows recognition of operators with unusually long spacers, deciphering the molecular basis of the arbitrium communication system.

## Introduction

Temperate bacteriophages can switch between their lytic and lysogenic life cycles. While this lytic-lysogeny selection is one of the key decisions in the biology of temperate phages, our understanding of the molecular mechanisms underpinning the decision-making process is still very limited, concentrating mainly on the model *Escherichia coli* λ phage (Golding, 2016). A novel decision-making system involved in phage induction/repression has been recently reported using the *Bacillus subtilis* phi3T phage as a model. In this elegant system, the phages produce a peptide (AimP) as a signal to communicate during phage infection (Erez et al., 2017). This system, termed “arbitrium,” seems to be used by a large group of SPbeta *Bacillus* phages and is composed of three genes: *aimP*, which encodes the arbitrium peptide, *aimR*, encoding a transcription factor that interacts with AimP, and *aimX*, which produces a small non-coding RNA that exerts a negative regulatory effect on lysogeny (Erez et al., 2017), therefore inducing the prophage and producing lysis by a mechanism that is not clearly understood. The active arbitrium peptide AimP is six amino acids (aa) long but is synthetized as a 43-aa pro-peptide that is secreted to the medium, processed, and internalized into the bacterial cell as the active AimP using the oligopeptide permease transporter. In the bacterial cytoplasm, the peptide binds to the AimR receptor and regulates its DNA binding activity (Erez et al., 2017). AimR is a transcription factor; in its apo peptide-free form it promotes *aimX* expression. In phi3T, the binding of AimP to AimR induces the dissociation of AimR active dimers producing inactive monomers that no longer promote *aimX* transcription. In the initial stages of infection, when the number of active phages is low, the arbitrium peptide is absent, and AimR activates *aim*X expression promoting lysis. During the lytic cycle, AimP will accumulate in the medium as a consequence of the excessive phage replication, until its concentration reaches the threshold level required to bind to its cognate AimR receptor (Erez et al., 2017). This important interaction promotes the switch to the lysogenic cycle and impairs the killing of the entire bacterial population by the phage. It has been hypothesized that AimR receptors from different phages are specifically regulated by their cognate arbitrium peptide, suggesting that phages only communicate with their progeny (Erez et al., 2017).

Although little is known about the molecular basis of the arbitrium system, it shares features with the RRNPP (Rgg, Rap, Npr, PlcR, and PrgX) family of quorum-sensing regulators (Declerck et al., 2007, Parashar et al., 2015). As in the arbitrium system, the RRNPP family is composed of a cytoplasmic receptor and a short (5–10 aa) signaling peptide produced by maturation of a pro-peptide (Neiditch et al., 2017). With the exception of the Rap subfamily, all RRNPP members present an architecture consisting of an N-terminal DNA-binding domain and a C-terminal peptide-binding domain. Structural characterization of different RRNPP members shows that peptide-binding domain consists of (5–9) tetratricopeptide repeats (TPR) that adopt a superhelical fold with a concave inner groove where the peptide is bound (Neiditch et al., 2017). Similarly to the arbitrium system, binding of the peptide to the TPR domain regulates the transcriptional activity of the RRNPP receptors. These similarities suggest that arbitrium systems are members of the RRNPP family with a similar mechanism of action, but phylogenetic analyses did not identify AimR as a new RRNPP family member (Neiditch et al., 2017). Therefore, it is not clear if the arbitrium system could be considered a new member of the RRNPP family or whether it represents a different unrelated quorum-sensing mechanism.

To shed light on the molecular basis of the arbitrium communication system, we have structurally and functionally characterized the system present in the prototypical SPbeta phage from *B. subtilis*. Initially, we solved the crystal structure of the SPbeta AimR receptor in its *apo* form, confirming that it has a similar architecture to the RRNPP family but with the particularity of a “break” TPR domain that brings plasticity to the protein. We determined that AimR dimers bind to an unusual operator composed of two 6-mer inverted boxes separated by a long 25 bp spacer. The AimR-DNA complex showed DNA recognition via canonical HTH domains while residues from the TPR domain interact with the spacer DNA backbone. Finally, we established the basis of this mechanism of communication by solving the structure of AimR-AimP complex. This structure shows how the peptide stabilizes a compact conformation of AimR that reduces the spacing between the DNA-recognition helices and prevents them from reaching the distal boxes but, surprisingly, keeps the DNA-recognition helices exposed in a DNA-binding competent conformation. These structures give clues about the mechanism of receptor-peptide selectivity, suggesting some promiscuity in this process. The competent conformation of AimR to bind DNA in its peptide-bound state and the plasticity of apo AimR that allows the protein to recognize the boxes with different sizes of spacers opens the possibility of additional regulatory activities of AimR and, consequently, suggests a more complex mechanism in the lysis-lysogeny decision regulated by the arbitrium system.

## Results

### AimR Displays a High Conformational Plasticity

**Figure 1:**
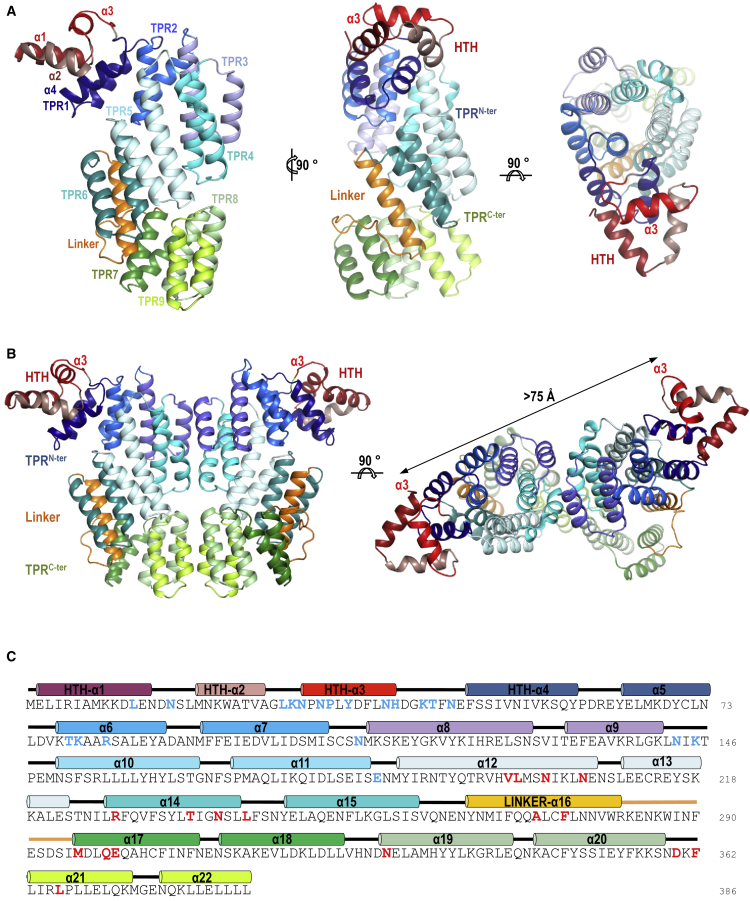
Structure of Apo SPbeta AimR (A) Three orthogonal views of one representative apo SPbeta AimR monomer in ribbon representation. The HTH DNA-binding domain is colored in different tones of red and the TPR-like motifs are colored in dark blue for TPR1, marine blue for TPR2, slate blue for TPR3, cyan for TPR4, light cyan for TPR5, turquoise for TPR6, dark green for TPR7, pale green for TPR8, and lime for TPR9. The linker connecting the TPR^N-ter^ and TPR^C-ter^ subdomains is colored in orange. (B) Representative dimer of the apo SPbeta AimR in ribbon representation. The structural elements are colored as in (A), and the DNA recognition helices α3 are labeled, and the distance between them in the dimer is indicated. (C) Sequence of SPbeta AimR. Structural elements are shown above the sequence colored as in (A). The residues interacting with the target DNA and AimP are highlighted in blue and red, respectively. See also Figures S1 and S3.

To establish the molecular basis of this novel mechanism of phage communication we produced crystals of AimR in its apo form that diffracted to 2.7Å and belong to space group P2_1_ with eight monomers in the asymmetric unit (Table 1). The overall structure of each monomer shows an N-terminal HTH DNA-binding domain (residues 1–59) directly connected (only 2 residues) to a C-terminal regulatory domain (residues 62–386) composed of 18 helices arranged in a two-layered right-handed superhelix (Figure 1A). The HTH motif includes 4 helices (helix α1–α4), and the predicted DNA recognition helix α3 is solvent exposed (Figure 1A), which is compatible with the active DNA-binding state proposed for the apo protein (Erez et al., 2017). Sequence analysis with TPRpred server (Zimmermann et al., 2018) only predicts the presence of four TPR, but the structure showed that the 18 α helices of C-terminal domain could be organized in 9 TPR-like motifs if helix α4 of the HTH domain was part of the first TPR (Figure 1A). These 9 TPR-like motifs (TPR1–9), unlike other members of the RRNPP family, can be further divided in two subdomains, one N-terminal (TPR^N-ter^; residues 46–281), which would include helix α4 and encompasses six TPR motifs (TPR1–6), and the other C-terminal (TPR^C-ter^; residues 295–386), including the remaining three TPR motifs (TPR7–9). A reminiscent TPR motif (residues 263–294), which has lost one of its helices, connects both TPR subdomains (Figure 1).

**Table 1:** Data Collection and Refinement Statistics

Data Collection	AimR Apo	AimR-DNA	AimR-AimP
**Space group**	**P2**_**1**_	**I4**_**1**_**22**	**P4**_**1**_**2**_**1**_**2**

Cell dimensions (Å)	a = 78.91, b = 251.20 c = 90.08 β = 90.89	a = b = 159.16 c = 245.91 a = β = γ = 90	a = b = 94.34 c = 249.95 α = β = γ = 90
Resolution (Å)	125.6–2.7 (2.84–2.7)^a^	133–2.2 (2.16–2.1)	89–2.3 (2.4–2.3)
Unique reflections	10,1047	79,845 (11,523)	51,207
Completeness (%)	100 (100)	100 (100)	100 (100)
Multiplicity	7.5 (7.7)	26.8 (27.9)	17.9 (14.7)
I/σ(I)	9.6 (1.6)	18.9 (2.6)	15.0 (1.8)
Rpim	0.07 (0.477)	0.025 (0.527)	0.037 (0.448)

**Refinement**			

R_work_	0.222 (0.336)	0.222 (0.334)	0.178 (0.272)
R_free_	0.252 (0.357)	0.251 (0.349)	0.218 (0.284)
Mean B factors (Å^2^)	47	48	48
Rmsd, bond (Å)	0.017	0.017	0.019
Rmsd, angles (°)	2.156	2.162	2
Monomers in ASU	8	2	2

**Ramachandran Plot**			

Most favored (%)	98.34	99.1	98.32
Additional allowed (%)	1.66	0.9	1.68

a Values in parentheses are for highest-resolution shell.

**Figure 2:**
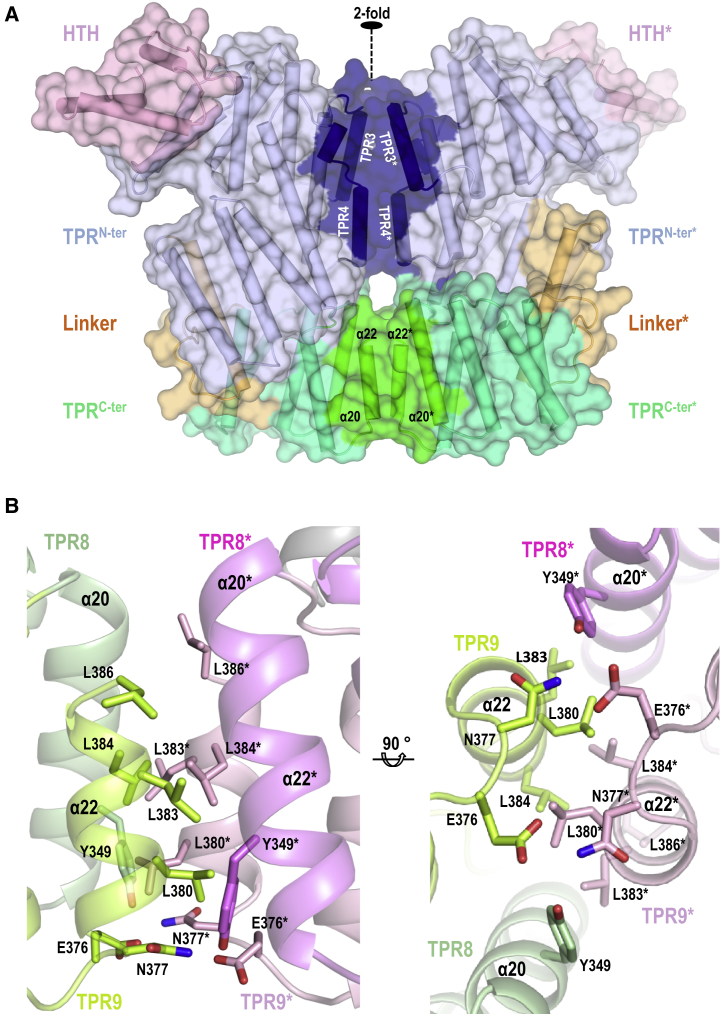
SPbeta AimR Dimerization (A) Overall structure of SPbeta AimR dimer in semitransparent surface over the protein in cartoon with the helices as cylinders with the HTH domain, the TPR^N-ter^ subdomain, the TPR^C-ter^ subdomain, and the linker colored in light pink, light blue, light green, and orange, respectively. Dimerization surfaces at the two TPR subdomains are highlighted in darker tones. The 2-fold dimerization axis is indicated. (B) Two orthogonal closed views of the two C-terminal TPR motifs forming a four-helix bundle that mediates the constant interface of dimerization. Helices are shown in cartoon in green tones for a monomer and pink tones for the other. Interacting residues are shown in stick with nitrogen and oxygen atoms colored in blue and red, respectively. TPR and residues are labeled, and an asterisk denotes that belong to the second monomer. See also Figures S2, S4, and S5.

The eight monomers (A through H) of the asymmetric unit are arranged into four stable dimers characterized by an overall interface area of around 1,250 Å^2^ (Figures 1B and S1). The dimeric organization in the apo state in solution was confirmed by using size-exclusion chromatography with multi-angle light scattering (SEC-MALS) (Figure S2A). Structural comparison showed that each AimR monomer adopts a slightly different conformation, suggesting intrinsic plasticity for this protein. Superimposed AimR monomers displayed rmsd values from 0.35 to 2.17 Å for 380 Cα atom pairs (Figure S3A). Protein domain motion analysis with Dyndom (Hayward and Berendsen, 1998) showed that this flexibility is due to the different relative disposition of two almost rigid portions, the first one including the HTH domain and the TPR^N-ter^, and the second one corresponding to the TPR^C-ter^ with a bending region (281–285) placed in the TPR connector that would work as structural hinge (Figures S3A and S3C). Interactions at the two ends of the TPR superhelix mediate AimR dimerization (Figure 1B). Detailed analysis of these two areas showed that one of them is conserved in all the dimers, but the second one is variable (Figure S4). The constant region of dimerization is nucleated by the interaction of the C-terminal α22 helices in TPR9, generating a helix bundle where also participate helices α20 from TPR8 that form a four-helix bundle (Figures 2A and 2B). The bundle has a hydrophobic core formed by the reciprocal interaction of residues L380, L383, L384, and L386 of C-terminal helix α22 and additional interactions mediated by E376 and N377 (Figure 2B). This hydroponic core is laterally shielded by the helices α20 that mediate contacts through residues Y349, I352, and K356. At the other end of the TPR superhelix is a second interface area defined by the interaction, mainly through hydrophilic contacts, of TPR3 (helices α9 and α10) and TPR4 (helix α11) motifs (Figures 2A and S4). Although these two TPRs are involved in all the AimR apo dimer interactions, the specific contact changes among dimers (Figure S4), indicating that this region works through a slipping interface of contact. As a consequence of this plasticity, the monomers acquire slightly alternative arrangements, producing a variation in the relative disposition into the dimer of the DNA recognition α3 helices (Figures S1 and S3). Thus, it is tempting to speculate that this plasticity might have a functional role for the DNA-binding activity of AimR.

To validate the leading role of the TPR^C-ter^ subdomain in the AimR dimerization, a mutant lacking this subdomain was generated by introducing a stop codon at position 294 (AimR^ΔC-ter^). As expected, size-exclusion chromatography analysis confirmed that AimR^ΔC-ter^ is monomeric in solution (Figure S2B). Comparison of AimR sequences from different phage families showed that the hydrophobic residues at the C-terminal helix are conserved (Figure S5), supporting the idea that dimerization through this structural element is a general feature for AimR receptors.

### AimR Receptor Recognizes an Unusual DNA Operator

**Figure 3:**
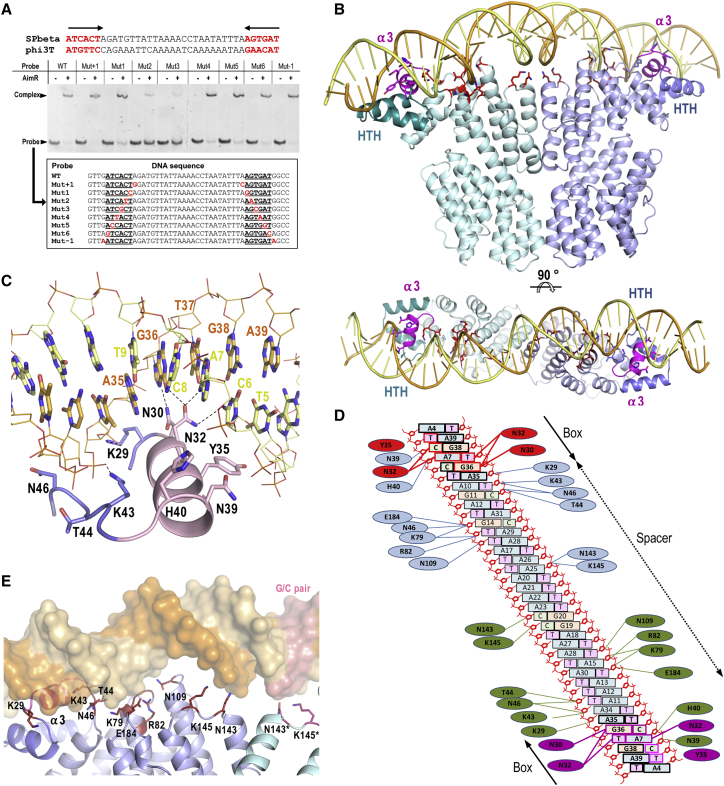
AimR Recognizes an Unusually Long DNA Operator (A) The DNA operator of SPbeta AimR identified in the footprinting experiments was confirmed by EMSA analysis. Sequences of DNA operators for SPbeta and phi3T AimR are shown in the top with the 6 bp inverted repeats highlighted in red. Representative EMSA with operators, including mutations in recognition boxes or in its boundaries with 1:10 DNA:protein molar ratio, is shown. The probes used in the EMSA analysis are shown in the table with the mutated bases highlighted in red. (B) Structure of SPbeta AimR bound to its DNA operator. Two perspectives of AimR dimer bound to a 43 bp oligonucleotide, including the DNA operator, are shown. AimR monomers are represented in ribbon and colored in cyan and blue. The HTH DNA-binding domains are highlighted in darker tones with the recognition helix α3 in magenta. The DNA interacting residues are shown in stick with carbon atoms colored in magenta and red for those involved in box and DNA backbone recognition, respectively. (C) Detail of DNA recognition by the helix α3. The helix α3 (light pink) is inserted in the major groove and recognizes the AimR box. Interacting residues are shown in sticks with carbon atoms colored as the corresponding structural element. Dotted black lines highlight polar interactions in the box readout. The bases are represented in stick with the same color as (B) and those bases corresponding to the box are labeled. (D) Schematic representation of the DNA-protein contacts. Sequence-specific contacts are highlighted with thicker lines and residues are colored in red and magenta for each monomer, respectively. Interactions with the backbone are colored in blue and green. (E) Interactions with DNA spacer. Details of the interaction between AimR and the 25 bp spacer are shown. The DNA is represented in semi-transparent surface with each strain in an orange tone. HTH and TPR residues interacting with the spacer backbone are shown in sticks and colored with carbons in red. Only one half of the operator is shown for clarity, with the double G/C pair at the middle of the operator highlighted in salmon. See also Figures S5 and S6, and Table S1.

It has been shown that AimR works as a transcriptional activator in its peptide-free state. Therefore, it was striking that in our crystal structure of apo AimR the DNA-binding helices α3 were separated more than 75 Å (Figure 1B). To determine whether SPbeta AimR resulted in a conformational change after DNA binding, or if, by contrast, we were facing an atypical conformation of the well-known HTH domains, we attempted to solve the crystal structure of SPbeta AimR in complex with its cognate promoter. It has been reported that the AimR protein from phi3T phage binds to a single DNA site downstream of the *aimP* gene. Thus, we analyzed the capacity of SPbeta AimR to bind to the downstream DNA region of SPbeta *yopL* gene, which would correspond to the phi3T *aimP* (Erez et al., 2017). Electrophoretic mobility shift assays (EMSA) confirmed that SPbeta AimR binds to a 359 bp fragment that encompasses the predicted binding region (Figure S6A). Footprinting analyses showed that AimR protects a 37 nt sequence from DNase I degradation (Figure S6B), which is composed of two perfect inverted repeats of the 6-bp ATCACT sequence separated by 25 bp (Figure 3A).

To confirm that SPbeta AimR recognizes the *ATCACTA*GATGTTATTAAAACCTAATATTTA*AGTGAT* operator, a serial set of double-stranded primers with punctual mutations in both palindromes or its boundaries were designed (Figure 3A and Table S1). EMSA assays showed that changes in the palindrome affect or abolish AimR binding, while changes in positions at the boundaries of the palindrome have a minor effect (Figure 3A). The most important effect was observed when changes were introduced at positions 4 and 5 (ATCACT) of the binding box, highlighting these as the key position for AimR binding (Figure 3A). The 25-bp spacer between binding boxes is unusually large but is compatible with the 75–90 Å separation between DNA-binding helices observed in the AimR apo structure. A sequence with similar organization, composed of two perfect inverted repeats of a 6-bp ATGTTC sequence separated by 25 bp was found in Phi3T phage downstream of *aimP* (Figure 3A). Binding of phi3T AimR to this sequence was confirmed by EMSA (Figure S6C), supporting that this operator architecture is common in AimR family.

### Structural Basis of DNA Recognition by AimR

To understand how AimR binds to its unusual operator sequence, we determined the crystal structure of SPbeta AimR bound to a 43-bp DNA fragment, including the AimR operator characterized here. SPbeta AimR-DNA crystals belong to space group I4_1_22 and diffracted to 2.2 Å resolution (Table 1). The asymmetric unit contains two AimR molecules arranged as a dimer and an operator-DNA molecule (Figure 3B). The overall structure and dimer arrangement is similar to the apo SPbeta AimR (Figures 1B and 3B). However, the relative disposition between the TPR^N-ter^ and TPR^C-ter^ subdomains is slightly different from that observed in the apo form, and this is reflected in rmsd differences when monomers from these structures are compared (rmsd from 0.92 to 2.06 Å), confirming the AimR plasticity. As a result of this flexibility, the HTH domain’s disposition changes and the DNA recognition helices α3 increase its separation (1–8 Å) with respect to the apo structures.

Similar to other HTH-type DNA binding proteins, the recognition helix α3 from the HTH motif inserts into the DNA major groove. Direct readout of the DNA is accomplished via the side chains of residues N30, N32, and Y35, which interact mainly with the T_5_C_6_A_7_/A_35_G_36_T_37_ motif of the SPbeta AimR box (Figures 3C and 3D). The N32 side chain establishes multiple hydrogen bonds recognizing C_6_ and A_7_ in one DNA strand and G_36_ and T_37_ in the other (Figures 3C and 3D). The G_36_ is hydrogen bonded to N30. Finally, the Y35 side-chain mediates hydrophobic interaction with the pyrimidine rings of T_5_ and C_6_ and hydrogen bonding with a phosphate of the DNA backbone (Figures 3C and 3D). Additionally, N30, N32, and P33 side chains mediate hydrophobic contacts with the TCA/AGT motif. Finally, the HTH domain also participates in the indirect readout of the operator DNA backbone through hydrogen bonds and Van der Walls contacts mediated by the side-chains of L12, N16, L28, K29, N30, N39, H40, K43, T44, and N46 (Figures 3C and 3D).

In addition, AimR interacts with the 25-bp spacer region. The K79 and R82 from helix α6 in TPR1 as well as N109, N143, and K145 placed in the loops connecting the helices of TPR2 and TPR3 interact with the DNA backbone (Figures 3D and 3E). These nonspecific interactions are distributed along the two DNA turns generated by the 25-bp spacer and help to maintain an extended conformation and induce some distortion in the DNA. Overall, the central axis for the DNA double helix is S-shaped, occurring at the S-twist in the middle of the spacer region where there is a double G/C pair flanked by two A/T-rich regions (Figures 3B, 3D, and 3E). The AimR binding also induces a small bend (around 20°) over the entire operator, with the concave side of the DNA facing the protein and the double G/C pair in the convex kink (Figures 3B and 3E). The sequence of the AimR operator from phage phi3T also showed an AT-rich spacer with only two G/C pairs but in this case separated by 6 bp (Figure 3A), suggesting that this architecture could be required to facilitate the recognition of distal boxes.

### Peptide Binding Site

**Figure 4:**
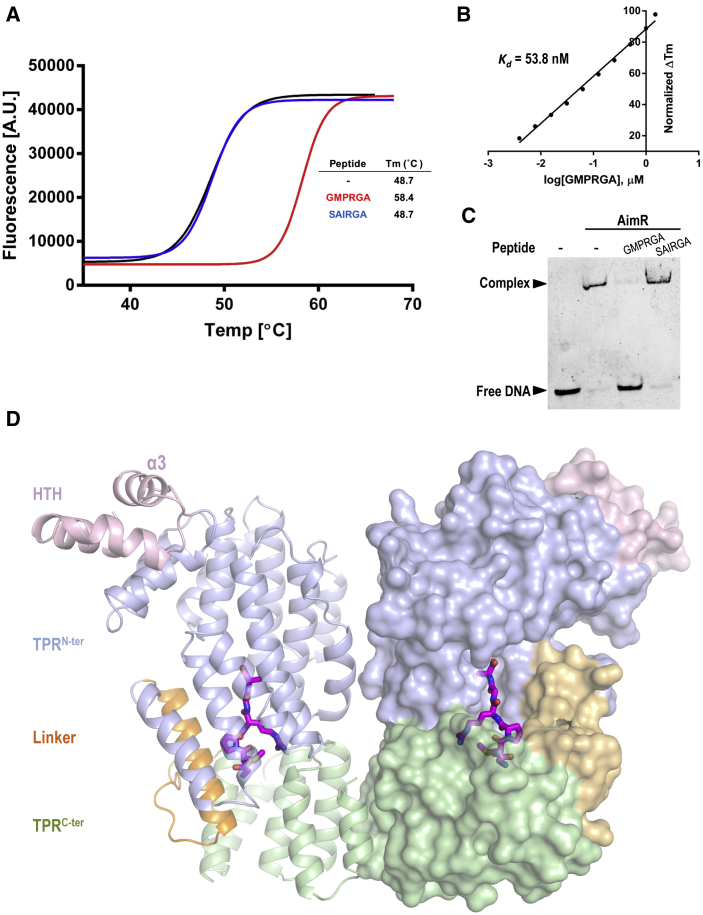
Binding of AimP to SPbeta AimR (A) Binding of the SPbeta-specific arbitrium peptide stabilizes the AimR protein. Thermal unfolding curves of AimR alone (black) or in presence of its specific arbitrum peptide (red) or the aribitrium arbitrium peptide of phi3T (blue). The curves and the calculated unfolding Tm from one of three independent experiments are shown. (B) Interaction of AimR with AimP measured by differential scanning fluorimetry. An experiment with 10 concentrations of peptide allows calculation of an apparent *K*_*d*_ of 53.8 nM. The data were fitted to a model for a single binding site per monomer. (C) EMSA showing that the SPbeta (GMPRGA), but not phi3T (SAIRGA), arbitrium peptide specifically disrupts the SPbeta AimR binding to its operator. (D) Structure of SPbeta AimR bound to AimP. The AimR dimer is represented with one monomer in semi-transparent cartoon in the other in semi-transparent surface. The peptide, represented in sticks with carbon atoms in magenta, is bound in the groove formed by the TPRs motifs. AimR HTH domain, TPR^N-ter^ subdomain, TPR^C-ter^ subdomain, and the linker are colored in pink, blue, green, and orange, respectively. See also Figures S3 and S4.

It has been proposed that the regulatory AimP peptide for SPbeta AimR has the GMPRGA sequence (Erez et al., 2017). To confirm the interaction with AimR, we performed thermal shift assays using the AimP peptide SAIRGA specific for phi3T phage as a negative control. The GMPRGA peptide induced a strong stabilization of SPbeta AimR, with an increment in the melting temperature (Tm) of ∼10°C (from 48.7°C to 58.4°C). No increment in the Tm was observed when the SAIRGA peptide was added (Figure 4A), suggesting that the stabilization induced by GMPRGA is specific. Titration of SPbeta AimR with GMPRGA by thermal shift assays estimated an apparent binding affinity for this peptide of 53.8 nM (Figure 4B). A similar binding affinity was estimated by microscale thermophoresis for the interaction of phi3T AimR with its cognate peptide SAIRGA (Erez et al., 2017). Finally, we checked by EMSA that addition of the GMPRGA peptide induced the release of AimR from DNA, while addition of the SAIRGA peptide had no effect, confirming AimR specificity (Figure 4C).

**Figure 5:**
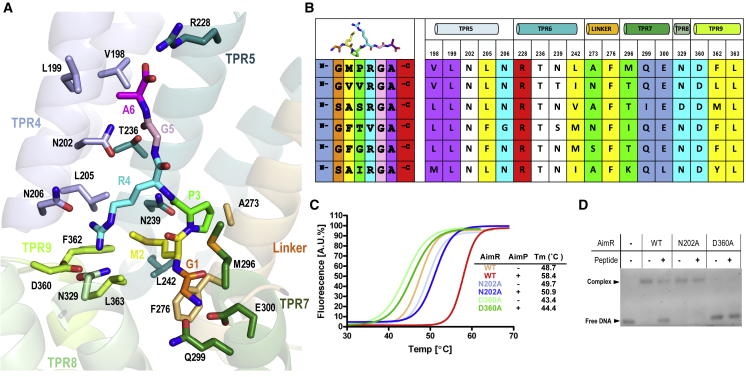
AimP Recognition by AimR (A) Close view of the arbitrium peptide binding site. The AimR structure is presented in semi-transparent ribbon and colored as in Figure 1. AimP interacting residues are shown in sticks, labeled, and colored with the carbon atoms as the corresponding structural element. AimP is shown in sticks, labeled, and colored with carbon atoms in orange (G1), yellow (M2), green (P3), cyan (R4), pink (G5), and magenta (A6). (B) Peptide recognition and specificity in AimR proteins. Arbitrium peptide (left) and the residues corresponding to the AimR positions interacting with the peptide in SPbeta (right) are aligned, showing a representative from each AimR major families. The sequence of SPbeta AimR and its AimP peptide are shown in the first line. A color code is used, showing with the same background color those residues of the peptide and AimR that interact. The colors for the peptide are the same as shown in (A), and the N and C-terminal end have been added with a background in blue and red, respectively. AimR residues interacting with peptide main chain are denoted with white background. At top are shown the structural elements where the AimR residues are placed. (C) Mutations in peptide anchoring residues abolish binding. Thermal unfolding curves of WT AimR and mutants N202A and D360A alone or in presence of AimP are shown. The curves and the calculated unfolding Tm from one of three independent experiments are shown. (D) EMSA assays with peptide binding mutants. The DNA-binding capacity and AimP inhibition were tested by EMSA for the AimR mutants N202A and D360A. WT AimR was used as a control. Notice that the AimR mutation D360A inhibits the capacity to bind to the AimR operator. See also Figures S2, S5, and S7.

Once we established GMPRGA as the AimP peptide for SPbeta AimR, we next attempted the crystallization of this complex in order to visualize the putative conformational changes induced by the peptide that preclude DNA binding. The crystals of the AimR-AimP complex diffracted up to 2.3 Å resolution in space group P4_1_2_1_2 containing two molecules of AimR in the asymmetric unit arranged as a dimer with one bound GMPRGA peptide per monomer (Figure 4D and Table 1). The peptide binds to the concave side of the channel formed by the TPR-like repeats of AimR (Figure 4D). The peptide is bound in an extended conformation that allows contact with residues from all of the TPR motifs with the exception of TPR1 (Figures 4D and 5A). This extended conformation is adopted by pulling hydrophilic interactions from both the N- and C-terminal ends. The peptide N-terminal amine is bound to Q299 and E300 side chains and to M296 main chain (TPR7), establishing hydrogen bonds and a salt bridge, while on the opposite end, the C-terminal carboxylate is salt bridged to R228 (TPR6) side chain (Figure 5A). The peptide lies in a hydrophobic cleft generated by the side chains of residues M92 (TPR2), Y159, L162, F167 (TPR4), V198, L199, L205 (TPR5), F232, L235, L242 (TPR6), I269, Q272, A273, F276 (linker), M296 (TPR7), M333 (TPR8), F362, and L363 (TPR9) and mediates van der Waals interactions with these residues both through its side and main chain (Figures 5A and S7). In addition, the AimP central main chain is anchored via direct hydrogen bonds of the oxygen atoms from peptide P3 and G5 with N202, T236, and N239 AimR side chains (Figures 5A and S7). AimP R2 side chain, which is highly conserved among arbitrium peptides, interacts by hydrogen bonds with N206 (TPR5) and N329 (TPR8) and a salt bridge with D360 (TPR8 and TPR9 loop) (Figures 5A and S7). The AimP M2 lies in the hydrophobic pocket generated by the side chains of AimR residues L205 (TPR5), N239, L242 (TPR6), F276 (linker), F362, and L366 (TPR9) (Figures 5A and S7). The pyrrolidine side chain of AimP P3 interacts with the side chains of A273 (linker) and M296 (TPR7). Finally, the Cβ carbon of AimP A6 is sandwiched between V198 and L199 from TPR5 (Figures 5A and S7).

### Arbitrium Peptide Recognition Specificity

Arbitrium peptides can be distributed in six major families (GMPRGA, GVVRGA, GFGRGA, SASRGA, GFTVGA, and SAIRGA), which monopolize 80% of the sequences (Erez et al., 2017). These peptides present two well-differentiated parts: a highly conserved RGA C-terminal half and a variable N-terminal region that should account for the observed peptide-receptor specificity. Therefore, anchoring of the conserved RGA motif to the receptor protein is expected to be mediated by conserved residues present in all AimR families. Comparison of protein sequences from representative AimR receptors of these major families supports this hypothesis. Thus, the peptide carboxyl end and main chain are fixed by the AimR fully conserved N202, R228, and T236 residues (Figure 5B). The Ala side chain is sandwiched between a couple of conserved hydrophobic residues, and the Arg is projected toward the TPR^C-ter^ subdomain to form a salt bridge with the strictly conserved D360 (Figure 5B). Conversely, variable residues in both partners, peptide and receptor, should provide specificity in the recognition. As it has been mentioned, the N-terminal portion confers variability in the peptide. In accordance, the AimR residues interacting with the third position of the peptide (A273 and M296 in SPbeta) are highly variable, especially at position 296 (Figure 5B). However, residues that recognize the second residue of the peptide present a lower variability. The second position of the peptide presents an invariable hydrophobic residue (M/V/F/A) and is recognized by a hydrophobic pocket formed by L205, L242, F276, F362, and L363. Sequence analysis showed the invariable presence of hydrophobic residues at these five AimR positions with F276 and L363 fully conserved (Figure 5B), suggesting that the combination of peptide side chain and receptor, and consequently steric limitation, should account for the specificity, although it could also allow some promiscuity. Finally, to ensure the proper conformation of the peptide that allows the readout by the receptor, the N-terminal variable portion of the peptide is anchored by interactions with highly conserved residues such as the N206 and E300 that recognize the peptide backbone and N-terminal end, respectively (Figure 5B).

To confirm these observations, N202 and D360 SPbeta AimR residues were mutated to Ala (AimR^N202A^ and AimR^D360A^), and their AimP binding capacities were checked by thermal shift assays. As expected for mutations that abolish peptide binding, addition of AimP only induces a residual stabilization (1° versus 10°in WT) in the mutant receptors (Figure 5C). SEC-MALS analysis showed that both AimR^D360A^ and AimR^N202A^ mutants are dimeric in solution, supporting the idea that the mutations had no major effect on protein folding (Figure S2C). EMSA assays confirmed AimR^N202A^ is able to bind DNA and addition of AimP did not induce DNA dissociation, confirming the loss of peptide binding capacity due to the mutation (Figure 5D). Remarkably, AimR^D360A^ did not bind to DNA, suggesting a structural role for D360. This observation is supported by the thermal shift assays where the AimR^D360A^ showed a decrease of 5.3°C in the melting temperature with respect to the WT AimR (Figure 5C). D360 is placed in helix α21 and contact residues of helix α20 essential in AimR dimerization, confirming, as has been observed in several members of RRNPP family, that peptide recognition residues mediated conformational changes that propagate along the protein (Gallego del Sol and Marina, 2013). Altogether, our analysis reveals that AimR receptors present a high number of conserved anchoring residues, which are involved in the conformational change that AimR suffers after AimP binding (see next section). Interestingly, the number of AimR residues involved in peptide specificity is limited, opening the door to crosstalk in lysis-lysogeny decision.

### Mechanism of Peptide Inhibition

It has been proposed that in phi3T, AimP blocks AimR function by inducing AimR dimer dissociation (Erez et al., 2017). Unexpectedly, SEC-MALS analysis confirmed that the addition of AimP did not induce SPbeta AimR dimer dissociation (Figure S2A), and the SPbeta AimR-AimP structure showed a dimer with a similar oligomeric organization to either the apo AimR dimer or the AimR dimer bound to its DNA, raising the question of how the peptide blocks AimR activity and suggesting a new mechanism of signal transduction for SPbeta AimR.

**Figure 6:**
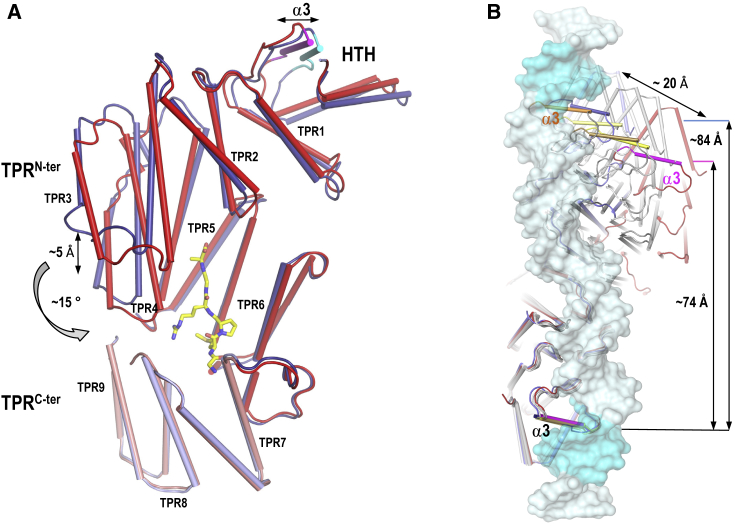
Conformational Changes in AimR upon AimP Binding (A) Superimposition of AimR structures from the AimP (red tones) and DNA (blue tones) complexes. The peptide (in sticks rendering with carbon atoms colored in yellow) induces an angular movement of approximation to the TPR subdomains. TPR3–TPR4 in TPR^N-ter^ subdomain are displaced toward TPR8–TPR9 in the TPR^C-ter^ subdomain, reducing the superhelical pitch and compacting the structure. The DNA recognition helices α3 are highlighted in magenta and cyan for AimP- and DNA-bound structures, respectively. Helices are shown as cylinders and the TPR motifs are labeled. (B) Peptide binding induces the displacement of the HTH binding domains. The structures of dimers of AimR in its apo form (four dimers colored in different tones of gray), AimP-bound (colored in red), and DNA-bound (colored blue) were superimposed at the level of one monomer (lower), and a zenithal view is shown. The DNA operator from the AimR-DNA structure is shown in semitransparent representation and colored in cyan with the binding boxes highlighted in a darker tone. The recognition helices α3 are highlighted in magenta and dark blue for the AimP- and DNA-bound structures, respectively. For the apo structures, the helices α3 are highlighted in different tones of yellow-orange. The displacement of helices α3 that produce a relative approaching of the HTH DNA binding domains in the AimP-bound structure is indicated. See also Figures S3 and S4.

To solve this mystery, we scrutinized the different solved structures. Interestingly, the structure of the apo SPbeta AimR showed intrinsic flexibility that affected the relative disposition of the HTH domains into the dimer (Figures S1A and S3A). A comparative analysis of the DNA- and AimP-bound forms of AimR explains how this plasticity is used by the peptide to regulate AimR activity. The AimP-bound form presents a more compact conformation, reflected in a reduction of the superhelix pitch of ∼5 Å with respect to the DNA-bound form (Figure 6A), and even bigger (∼11 Å) with respect to some apo forms. The closure movement induced by AimP binding (between 10° and 20° calculated with Dyndom [Hayward and Berendsen, 1998]) reduces the distance between the TPR^N-ter^ and TPR^C-ter^ subdomains (Figure 6A), allowing new interactions between residues of both subdomains that stabilize the peptide-bound conformation. Superimposition of the two monomers present in AimR-AimP dimer showed that both subunits are almost identical. Oppositely, clear differences exist for the DNA-bound and the apo forms between subunits (Figure S3), supporting that AimP stabilizes the protein in a closed conformation induced by the interaction of N- and C-terminal peptide ends with TPR^C-ter^ and TPR^N-ter^, respectively, which force the approach of both subdomains to reach these interactions (Figures 5A and 6A). Furthermore, it is reinforced by the cross-interaction of the R4 and M2 peptide side-chains with residues of both AimR subdomains (Figure 5A). Interactions with peptide ends and conserved R4 involve salt bridges with strictly conserved residues in AimR receptors (Figure 5B), indicating that this type of conformational change should be general for this family of proteins. The participation of a variable residue (M2) in the induction and stabilization of the closed conformation imposes peptide-receptor selectivity in the movement. It is worth noting that AimR closing is a rigid body movement since N- and C-terminal portions of the protein are almost identical in all the conformations (either AimP-bound or DNA-bound or apo) (Figure S3), with the linker region acting as a hinge. Sequence analysis showed that the linker is highly conserved among the AimR receptors (Figure S5), so it must be a key structural element in the mechanism of signal transduction induced by AimP.

Obviously, the closing movement of the monomers severely impacts the architecture of the dimer, unraveling the mechanism of action of the arbitrium system. Comparison of AimP and DNA-bound structures confirms that the closed conformation induced by AimP reduces the distance between the DNA recognition helices (Figure 6B). As a consequence, AimP fixes an AimR conformation unable to reach the two distant half sites of the AimR operator. DNA binding-domain reorientation is facilitated by a slipping dimerization interface, precluding a productive disposition to recognize the target operator. Surprisingly, the inhibited conformation induced by AimP still disposes the DNA recognition helices exposed to the solvent in a competent conformation to bind DNA (Figures 4D and 6B), suggesting the intriguing possibility that other genes could be regulated by this receptor in the presence of AimP.

### AimR Plasticity Allows Recognition of Variable-Length Spacer Operators

**Figure 7:**
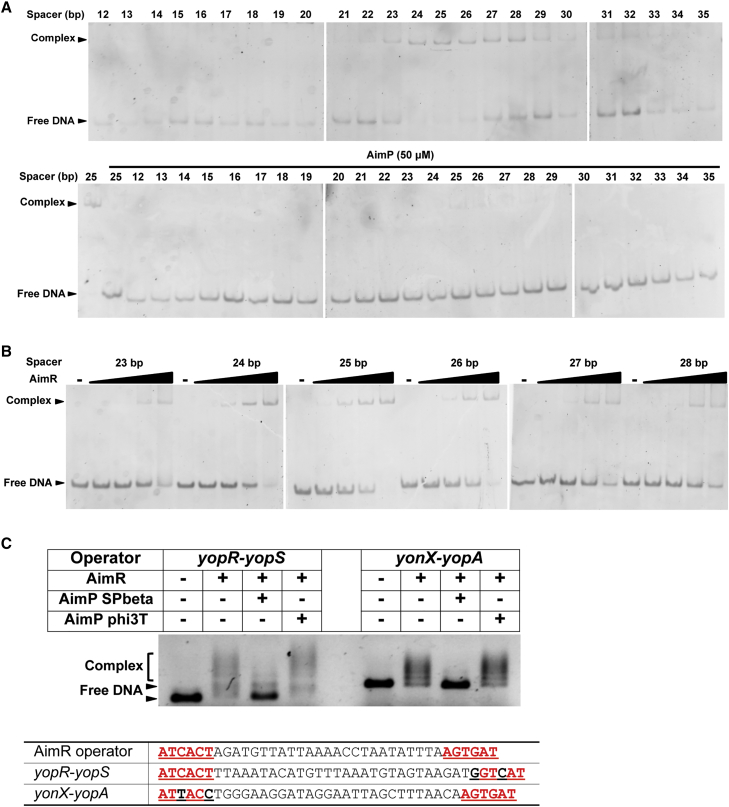
Spacer Length Effect in AimR Operator Recognition (A) EMSA analysis with DNA operators presenting spacers from 12 bp to 35 bp and SPbeta AimR in its apo form (upper) or in the presence of AimP (lower). (B) Titration assays of spacer-length-variant operators with increased amount of SPbeta AimR (from 0.125 to 1 μg). (C) EMSA analysis of two putative AimR operators found in SPbeta genome with alternative spacer length and recognition sequence degeneracy. Specificity was checked by the capacity of SPbeta but not phi3T AimP peptide to inhibit the DNA binding. Sequence of each putative operator as well the AimR operator are shown, with the inverted repeats highlighted in bold red letters. See also Tables S1 and S2.

We explored the possibility, suggested by our structures, that AimR or the AimR-AimP complex could bind to the DNA inverted repeats separated by longer or shorter spacers. To do this, we performed EMSA using a battery of operators, where the spacer length was decreased base to base to 12 bp (−13 bp with respect to the original AimR operator) or increased to 35 bp (+10 bp). These experiments showed that the apo AimR conserves its capacity to bind operators with either shorter or longer spacers (Figure 7A). The reduction of the spacer seems to have a more deleterious effect, since after eliminating 2 bp AimR dramatically loses its capacity to bind the operator. Conversely, AimR can recognize operators in which the spacer has been increased even more than 6 bp (Figure 7A). Titration assays showed that AimR presents a similar affinity for operators with spacers between 23 and 28 bp, decreasing the affinity, although very gradually, as the size of the spacer increases over 28 bp (Figure 7B).

In contrast, the AimR-AimP complex is unable to bind DNA, even to operators with spacers whose size (19–22 bp) would correspond to the separation of the recognition helices in the AimR-AimP complex (∼75 Å), indicating that the rigidity imposed by the peptide prevents binding (Figure 7A). These results confirm that the plasticity showed by apo AimR is essential for DNA binding and at the same time allows a high degree of promiscuity in the operator recognition, opening the door to additional regulatory functions. To confirm this hypothesis, we analyzed the SPbeta genome and found more than ten operators with similar boxes to those recognized by AimR separated by spacers of variable lengths (Table S3). Although the boxes in these operators present some degeneracy, they preserve the key AC pair at positions four and five. EMSA assays using two of these operators presenting spacers of 27 and 28 bp, respectively, confirmed our hypothesis (Figure 7C). Remarkably, the observed binding is specific since addition of the SPbeta AimP, but not the phi3T AimP, induces DNA release (Figure 7C).

## Discussion

The functional characterization of the arbitrium system suggested a mechanism of action reminiscent of the quorum-sensing RRNPP family (Erez et al., 2017). This family is exclusive to the *Firmicutes*, and interestingly the arbitrium system has been found uniquely in temperate prophages infecting this bacterial group (Erez et al., 2017, Neiditch et al., 2017). This correlation clearly suggests that phages of the SPbeta group have acquired all the elements required to communicate through the arbitrium system from their host bacteria. This elegant strategy allows them to parasitize the bacterial machinery during the communication process for their own benefit. This tactic is not unique to temperate prophages, and other mobile genetic elements present in the *Firmicutes*, such as integrative-conjugative elements (ICEs), also use communication systems of the RRNPP family to regulate their propagation in nature (Auchtung et al., 2005, Chandler and Dunny, 2004). This highlights the idea that peptide-mediated communication plays a key role in the ecology and evolution of this bacterial group.

However, phylogenetic analysis did not identify the transcriptional regulator AimR as a member of the RRNPP family. This contradicted the *in silico* structure-prediction analyses, which clearly suggested that AimR is likely to be a member of this family (Neiditch et al., 2017). With this discrepancy in mind, we aimed to solve this mystery. Our SPbeta AimR structures confirm the close relationship between the arbitrium receptor AimR and the quorum sensor receptors of the RRNPP family. Structural homology searches with PDBeFold (Krissinel and Henrick, 2004) and Dali (Holm and Laakso, 2016) servers using AimR as a query confirmed the structural homology with different RRNPP family members, in particular with the Rap group (Gallego del Sol and Marina, 2013, Parashar et al., 2013) (rmsd 3.2–3.4 Å for 305 aa aligned), which includes the peptide receptor of the aforementioned ICEs (Auchtung et al., 2005). These structural homologies also reflect functional similarities in the mechanism of action of AimR and other RRNPP members. However, the arbitrium system has some interesting features as a consequence of its adaptation to the phage life cycle (see below). Since our results have clearly demonstrated that AimR is a bona fide member of the RRNPP family, we propose to rename the family as RRNPPA (Rgg/Rap/NprR/ PlcR/PrgX/AimR), to include the names of all members.

The structure of AimR in different functional states reveals specific characteristics of the arbitrium system. The AimR receptor has particularly intrinsic flexibility, with the monomers in the apo state eliciting a “chewing-like” movement produced by the alternative relative disposition between its N- and C-terminal portions (Figure S3A). Thus, in its apo form, AimR is sampling multiple conformations in order to recognize its target DNA operator (Figure 6B). We have characterized the AimR operator, which has two perfect-inverted sequences separated by an unusual 25-bp-long spacer. Analysis of other structures available in the PDB revealed that no other protein-DNA complex deposited presents similar architecture. Therefore, this long spacer, which introduces mobility at the two distal binding boxes, correlates with the plasticity showed in the apo AimR. The structure of AimR bound to its operator confirms a canonical recognition of the palindromic boxes by the HTH domain. Thus, the size of the spacer is not related to any special requirement for DNA recognition. Remarkably, the presence of identical operator structures in arbitrium systems from other phages supports the biological relevance of this novel organization. But why?

The AimP-bound structure of AimR answered this question and showed a locked conformation where the HTH recognition helices approach one another (Figure 6B). This conformational change prevents them from reaching both distal boxes simultaneously, explaining the inhibitory effect provoked by the peptide. Surprisingly, in the inhibited conformation the AimR recognition helices remain exposed in a competent DNA-binding conformation (Figure 6B). However, we were unable to find an operator region supporting binding to the AimP-AimR complex. This could be easily explained because the rigidity imposed by the peptide impairs the recognition of the boxes in the new relative disposition after spacer reduction (with a relative rotation of the boxes of 90°–120°). We cannot discard, however, that the AimP-AimR complex could recognize operators with alternative boxes. Remarkably, the plasticity displayed by the apo AimR form allows the binding to new cognate operators in which the DNA-binding sites are separated by more or fewer base pairs, supporting again the idea that AimR control additional phage genes. In fact, we demonstrated AimR binding to other SPbeta operators with longer spacers. Based on these results, it is tempting to speculate that regulation of multiple phage and/or bacterial genes by a unique regulator (AimR) is an elegant strategy of molecular piracy.

Our results have established the mechanism of action of this peptide-induced inhibition and revealed the molecular basis of the peptide-receptor specificity in the arbitrium system. Arbitrium regulatory peptides have a clear polarity in their sequence identity; while the C-terminal residues are highly conserved among AimP peptides, the N-terminal residues are more variable. On the other hand, AimR residues involved in C-terminal AimP binding are conserved among AimR receptors. This implies that AimR has a primary and conserved design, which allows anchorage of the peptide in a competent conformation to induce the locked state. AimP anchoring is mainly produced by the interaction of both peptide ends with opposite poles of the AimR binding groove, together with recognition of the peptide main chain. In all the members of the RRNPP family, a conserved Asn residue is involved in peptide backbone contacts (Gallego del Sol and Marina, 2013, Grenha et al., 2013, Makthal et al., 2016, Parashar et al., 2015, Shi et al., 2005, Zouhir et al., 2013), and our structure confirms this anchoring mechanism for the AimR-AimP interaction. AimR N202 and N239 interact along the longitudinal axis of AimP and maintain its extended conformation. Recognition of the peptide ends has a major mechanistic impact since it induces and stabilizes the closed conformation pulling of both TPR subdomains. This interaction is mediated by salt bridges with conserved AimR residues. In addition, a conserved Arg at the fourth position of AimP secures the locked conformation by cross-contact of its side chain with AimR conserved residues present in both TPR subdomains. Conversely, AimR peptide-recognition residues showing variability among receptors are restricted to fewer positions that are key in the peptide-receptor selectivity. However, the existence of AimR variants, encoded by other phages, having similar residues in the peptide-recognition domain (e.g., hydrophobic residues that could interact with the second peptide position) suggests that the AimR receptors could be promiscuous in their ability to interact with different AimP peptides. This observation, together with the fact that AimP peptides present a conserved C-terminal region, suggests the existence of crosstalk between phages that use the arbitrium system, but the initial studies using the phi3T and SPbeta AimR receptors did not support this hypothesis (Erez et al., 2017). We propose here, however, that the SPbeta and phi3T phages used to test this hypothesis were not the most appropriate model. The AimP peptides recognized by these phages, GMPRGA (SPbeta) and SAIRGA (phi3T), have a completely different sequence in their variable regions. Moderate changes of one or two positions within some peptides are more common among AimPs. In some cases, these changes are extremely conservative, uniquely affecting a single position (M by F or I by V). Therefore, the crosstalk could have evolved to allow communication with closer “relatives” (including their own phage progeny). If true, this would represent a fascinating mechanism of phage altruism by promoting the survival of other members of the family. In agreement with this proposition, permissiveness in peptide selectivity, and crosstalk between related strains has been described in other members of RRNPP family (Fontaine et al., 2013, Perchat et al., 2011).

The crystal structures of the SPbeta AimR alone and in complex with AimP have been published during the submission of this article by different groups (Wang et al., 2018, Dou et al., 2018, Zhen et al., 2018), providing both corroboratory and complementary views of AimR plasticity and AimP binding. In the structures of SPbeta present in these publications, the DNA-binding HTH domains were traced with difficulty or were not directly observable, supporting the plasticity of AimP but hampering a deep mechanistic deduction. Additionally, differences with the structures presented here are observed in the oligomerization state. Although all the AimR structures present a dimeric state, the important interaction between the TPR3 and TPR4 motifs in the slipping dimerization region is not observed in the structures presented by other groups. We hypothesize that this difference is due to the presence of a C-terminal His-tag in the structures presented by the other groups, which is placed close to the structural elements involved in protein dimerization and could interfere in the dimer organization. In fact, the previously reported structures do not show any conformational change upon peptide binding, limiting the mechanistic information that can be deduced about the peptide inhibition. However, these structures are highly valuable to reveal the clues of AimP recognition, which are also in accordance with our structures. In summary, while these previous structures describe how the AimR receptor recognizes AimP, the present manuscript provides important mechanistic insights into the understanding of this novel regulatory pathway by providing the molecular mechanism of peptide inhibition, by providing the structure of AimR in complex with its cognate DNA, and by characterizing the organization of the unusual AimR-DNA binding operator. These results are crucial to fully understanding how AimP controls AimR activity, thus promoting phage lysogeny in nature.

## STAR★Methods

### Key Resources Table

**Table:** 

REAGENT or RESOURCE	SOURCE	IDENTIFIER
**Bacterial and Viral Strains**		

*Escherichia coli* BL21 (DE3) codon plus RIL	Agilent	Cat#230245
*Bacillus subtilis* 168	BGSC	BGSC 1A1

**Chemicals, Peptides, and Recombinant Proteins**		

GMPRGA (95% purity) peptide	ProteoGenix	N/A
SAIRGA (95% purity) peptide	ProteoGenix	N/A
Dream taq DNA polymerase	Thermo Scientific	Cat#10160790
LB medium	Fisher Scientific	Cat#BP1426-2
M9 minimal medium	Molecular Dimensions	Cat#MD12-501
L-selenomethionine	Fisher Scientific	Cat#10553601
DNase I	Sigma-Aldrich	Cat#D4263-1VL
TEV protease	Alberto Marina Lab	N/A
Sypro Orange	Sigma-Aldrich	Cat#S5692-50UL
RedSafe Nucleic Acid Staining Solution	Intron	Cat#21141
Crystallization screenings JBS I, JBS II	Jena Biosciences	Cat#CS114-L
Crystallization screening JCSG	Molecular Dimensions	Cat#MD1-40

**Critical Commercial Assays**		

Q5 Site Directed Mutagenesis Kit	NEB	Cat#E0554
ExoProStar PCR cleanup kit	Sigma Aldrich	GEUS79050

**Deposited Data**		

Atomic coordinates of Apo AimR	This paper	6HP3
Atomic coordinates of AimR-AimP complex	This paper	6HP5
Atomic coordinates of AimR-DNA complex	This paper	6HP7
Original data in Mendeley dataset	This paper	https://doi.org/10.17632/nytymgbhfm.1

**Oligonucleotides**		

AimR Phi3T synthetic gene	Biomatik	N/A
ssDNAoligos (see Table S1 for sequences)	Macrogen	N/A
dsDNAoligos (see Table S1 for sequences)	Macrogen	N/A

**Recombinant DNA**		

pLIC-SGC1	Nicola Burgess-Brown lab	Addgene plasmid # 39187

**Software and Algorithms**		

COOT	Emsley and Cowtan, 2004	https://www2.mrc-lmb.cam.ac.uk/personal/pemsley/coot/
Crank (CCP4 suported program)	Pannu et al., 2011	http://www.ccp4.ac.uk/html/crank.html
Dyndom	Hayward and Berendsen, 1998	http://dyndom.cmp.uea.ac.uk/dyndom/
GraphPad prism	GraphPad Software	https://www.graphpad.com/scientific-software/prism/
Phaser (CCP4 supported program)	McCoy et al., 2007	http://www.ccp4.ac.uk/html/phaser.html
Refmac (CCP4 supported program)	Vagin et al., 2004	http://www.ccp4.ac.uk/html/refmac5.html
Scala (CCP4 supported program)	Evans, 2006	http://www.ccp4.ac.uk/html/scala.html
XDS	Kabsch, 2010	http://xds.mpimf-heidelberg.mpg.de/html_doc/downloading.html

### Contact for Reagent and Resource Sharing

Further information and requests for reagents should be directed to Lead Contact Alberto Marina (amarina@ibv.csic.es).

### Method Details

#### Protein production and purification

The *aimR* gene from the SPbeta phage was amplified using primers PlicAimR+ and PlicAimR- (Table S1) and genomic DNA from *Bacillus subtilis* strain 168 as template. The PCR product was purified and cloned into the pLicSGC1 plasmid using Ligation-Independent Cloning (LIC) system. The resulting plasmid expresses the full-length AimR (residues 1-386) with an N-terminal 6xHistag followed by a TEV protease cleaving site. The protein was expressed using *E. coli* strain BL21 (DE3) RIL (Agilent). A single colony carrying the expression plasmid was grown overnight at 37°C in 100 ml of LB medium supplemented with 100 μg/mL of ampicillin and 33 μg/mL of chloramphenicol. The culture was used to inoculate 4 L of LB medium (dilution 1:100) containing ampicillin and chloramphenicol and was grown until cells reached an OD at 600 nm of 0.4. Then, the temperature was set to 20°C and a final concentration of 0.2 mM of IPTG was added. The culture was incubated at 20°C for additional 16 h. Cells were harvested by centrifugation and the pellet was suspended in lysis buffer (25 mM Tris-HCl pH 8, 250 mM NaCl) and lysed by sonication on ice. Cell debris was removed by centrifugation at 10.000 g for 1 h. The supernatant was loaded onto a 5 ml HisTrap FF (GE Healthcare) column, washed and eluted with lysis buffer supplemented with 500 mM imidazole. Fractions containing the purest protein were pooled and digested with TEV protease (50:1 molar ratio protein:TEV) and dialyzed against lysis buffer. The sample was concentrated and loaded in a Hi-Load Superdex 200 16/60 (GE Healthcare) gel filtration column equilibrated in lysis buffer. The purest fractions judged by SDS-PAGE were pooled, concentrated at 100 mg/mL and stored at −80°C. Typical yields were 35 mg recombinant protein/L of culture medium. Selenomethionine (SeMet) derivative SPbeta AimR was obtained by growing cells in M9 minimal medium (purchased from Molecular Dimensions) supplemented with L-selenomethionine (30 mg/mL) as well as amino acids inhibiting methionine synthesis (isoleucine, leucine and valine at 50 mg/l; lysine, phenylalanine and threonine at 100 mg/l). Purification protocol for SeMet labeled protein was identical to the native protein. The *aimR* gene from phi3T phage was ordered as a synthetic gene cloned in pET3a plasmid (Biomatik) with an N-terminal 6xHistag. The protein was expressed and purified following the same protocol used AimR from SPbeta but lower yields were obtained (∼5 mg recombinant protein/L of culture medium).

#### Protein Crystallization and data collection

The crystals were grown as hanging drops at 21°C with a vapor-diffusion approach. Initial crystallization trials were set up in the Cristalogenesis service of the IBV-CSIC using commercial screens JBS I, II (JENA Biosciences) and JCSG+ (Molecular Dimensions) in 96-well plates. Initial hits were reproduced and improved using the sitting drop method mixing equal volumes of protein at 10 mg/mL and homemade solutions. The apo form of SPbeta AimR crystallized in 15% PEG4000, 0.1 M MES pH 6.5 and 0.55 M NaCl. The AimR-AimP complex was prepared by adding 1mM of the GMPRGA peptide to the protein solution and using 1.5 M Ammonium sulfate as crystallization solution. AimR-DNA complex were obtained in 30% PEG400, 100 mM HEPES pH 7.5, 200 mM MgCl_2_ by mixing equal volumes of mother liquor and AimR:double stranded DNA (TTGATCACTAGATGTTATTAAAACCTAATATTTAAGTGATGGC) at 2:1 molar ratio. All type of crystals grew in 2-3 days and were directly flash frozen in liquid nitrogen. Diffraction data was collected from single crystals at 100°K on ALBA (Barcelona, Spain) and DLS (Didcot, UK) synchrotrons. Single wavelength dataset of the SetMet derivative crystals of AimR-peptide complex was collected at the Se peak calculated from fluorescence scanning on XALOC beamline (ALBA synchrotron). Datasets were processed with XDS (Kabsch, 2010) and reduced using Scala (CCP4) (Evans, 2006). The data-collection statistics for the best datasets used in structure determination are shown in Table 1.

#### Phase determination, model building and refinement

The AimR-peptide structure was determined by Single-Wavelength Anomalous Dispersion (SAD) using the data from the SeMet derivative. Crank (CCP4) (Pannu et al., 2011) was used to locate heavy atoms, calculate and extend phases and to build the initial model. The final model was generated by interactive cycles of manual model building using COOT (Emsley and Cowtan, 2004) and computational refinement with Refmac (Vagin et al., 2004). The structures of apo AimR and AimR-DNA complex were determined by molecular replacement using Phaser (McCoy et al., 2007) and the coordinates of AimR-AimP complex as search model. Data collection and refinement statistics are summarized in Table 1.

#### EMSA assays

AimR binding to its operator and the inhibition induced by the arbitrium peptides were analyzed by native polyacrylamide and agarose gel electrophoresis. DNA probes were amplified by PCR using primers showed in Table S1 and PCR products were purified. Double strand DNA primer probes were purchased from Macrogen (Table S1 and Figure 3A). Purified PCR product or purchased probes (0.4μM) and AimR (4μM) protein were mixed in EMSA buffer (50 mM HEPES pH 7.5, 5 mM MgCl_2_, 75 mM NaCl and 0.5 mM EDTA). The samples were incubated for 10 min at room temperature. 8% polyacrylamide gels were electrophoresed in Tris-Borate-EDTA (TBE) buffer at 100V for one hour, followed by loading of the samples. Electrophoresis was then performed in TBE buffer at 4°C for about 150 min at 100V. Agarose gels (0.8%–1.2%) were run in TAE buffer at room temperature for about 100 min at 80V. Gels were stained with RedSafe (Intron Biotechnology).

#### Nuclease protection assay

A 359 nucleotide fragment from the intergenic region between *yop*L and *yop*M was amplified using primers YopL/YopM+ and YopL/YopM- (Table S1) with one of the primers labeled with a fluorescein. PCR product was treated with ExoProStar™ kit (Sigma-Aldrich) and separated in two samples, using one as a control and incubating the second one with 0.3 μM AimR SPbeta for 10 min at room temperature. Digestion with DNase I (Sigma-Aldrich, 10^−4^ mg/mL) was performed for 1 min at 37°C in DNase I buffer. The reaction was stopped by adding EDTA to a final concentration of 10 mM and incubating at 80°C for ten minutes. The fragments generated by the digestion were analyzed by capillary electrophoresis in a ABI 3500 Genetic analyzer (Applied Biosystems) at the DNA sequencing service of the Instituto de Biomedicina de Valencia, and the chromatograms of the two samples (control and AimR treated) were compared searching for protected zones.

#### Thermal shift assay

The thermal shift assay was conducted in a 7500 Fast Real time PCR System (Applied Biosystems). Samples of 20 μL containing 5 × Sypro Orange (Sigma-Aldrich) and 20 μM of protein in a 20 mM Tris pH 8 and 250 mM NaCl buffer were loaded in 96-well PCR plates. To calculate the apparent *K*_*d*_ peptide concentrations from 0 to 2 μM were added to the mixture. Samples were heated from 20 to 85°C in steps of one degree. Fluorescent intensity was plotted versus temperature and integrated with GraphPad Prism software using a Boltzmann model to calculate melting temperatures.

#### Size Exclusion Chromatography with Multi-Angle Light Scattering (SEC-MALS)

SEC-MALS experiments were performed using an AKTA pure system (GE Heralthcare) coupled to a Wyatt DAWN HELEOS-II MALS instrument and a Wyatt Optilab rEX differential refractometer (Wyatt). 50 μL of the protein samples were injected at a concentration of 5 mg/mL on a Superdex 200 HR 10/300 (GE Heralthcare) column equilibrated in 25 mM Tris pH 8, 250 mM NaCl. When the effect of the peptide in the oligomeric state was tested, 1 mM peptide was added to the injected sample and the running buffer was supplemented with peptide at a final concentration of 1 μM. The Astra 7.1.2 software from the manufacturer was used for acquisition and analysis of the data.

### Data and Software Availability

Coordinates for atomic structures have been deposited at the RCSB Protein Data Bank (PDB: 6HP3, 6HP5, 6HP7).

## Supplemental Information

Document S1. Figures S1–S7 and Tables S1 and S2

Document S2. Article plus Supplemental Information
