# Supplementary material for: Deciphering the Molecular Mechanism Underpinning Phage Arbitrium Communication Systems
Source: Mol Cell. 2019 Apr 4;74(1):59–72.e3. doi: 10.1016/j.molcel.2019.01.025 (PMC6458997; doi:10.1016/j.molcel.2019.01.025)
Supplement: Document S1. Figures S1–S7 and Tables S1 and S2 [file mmc1.pdf]

Molecular Cell, Volume *74*

Supplemental Information

Deciphering the Molecular Mechanism Underpinning Phage Arbitrium Communication Systems

Francisca Gallego del Sol, José R. Penadés, and Alberto Marina

Supplementary Information

Deciphering the molecular mechanism underpinning phage arbitrium communication systems

Francisca Gallego del Sol, José R Penadés, Alberto Marina.

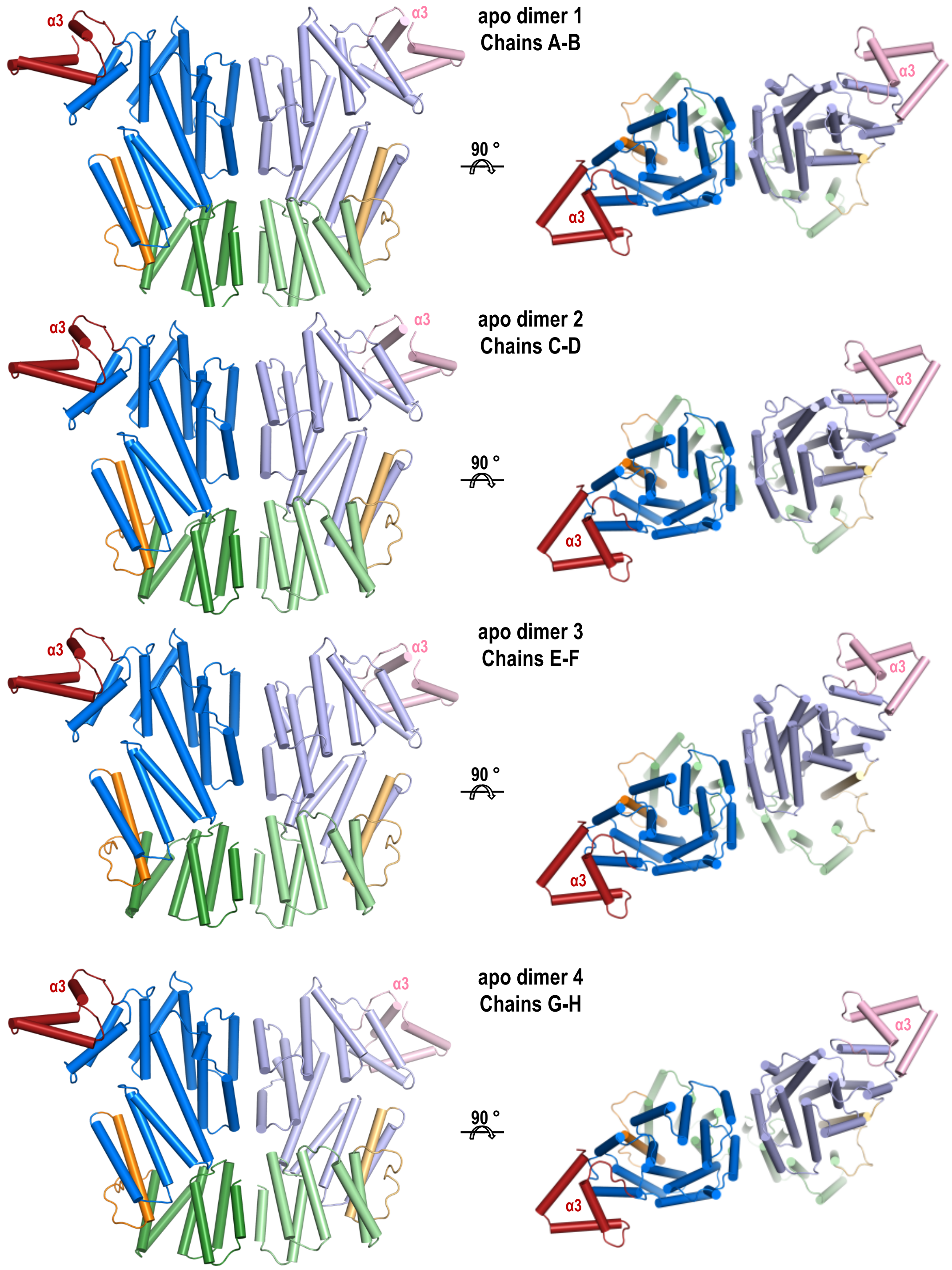

Figure S1. Structures of SPbeta AimR in apo state. Related to Figure1. Cartoon representation of the four dimers of SPbeta AimR in their apo state. The HTH domain, $TPR^{N\text{-}ter}$ and $TPR^{C\text{-}ter}$ subdomains and the linker are colored in pink, blue, green and orange (different tones for each monomer), respectively.

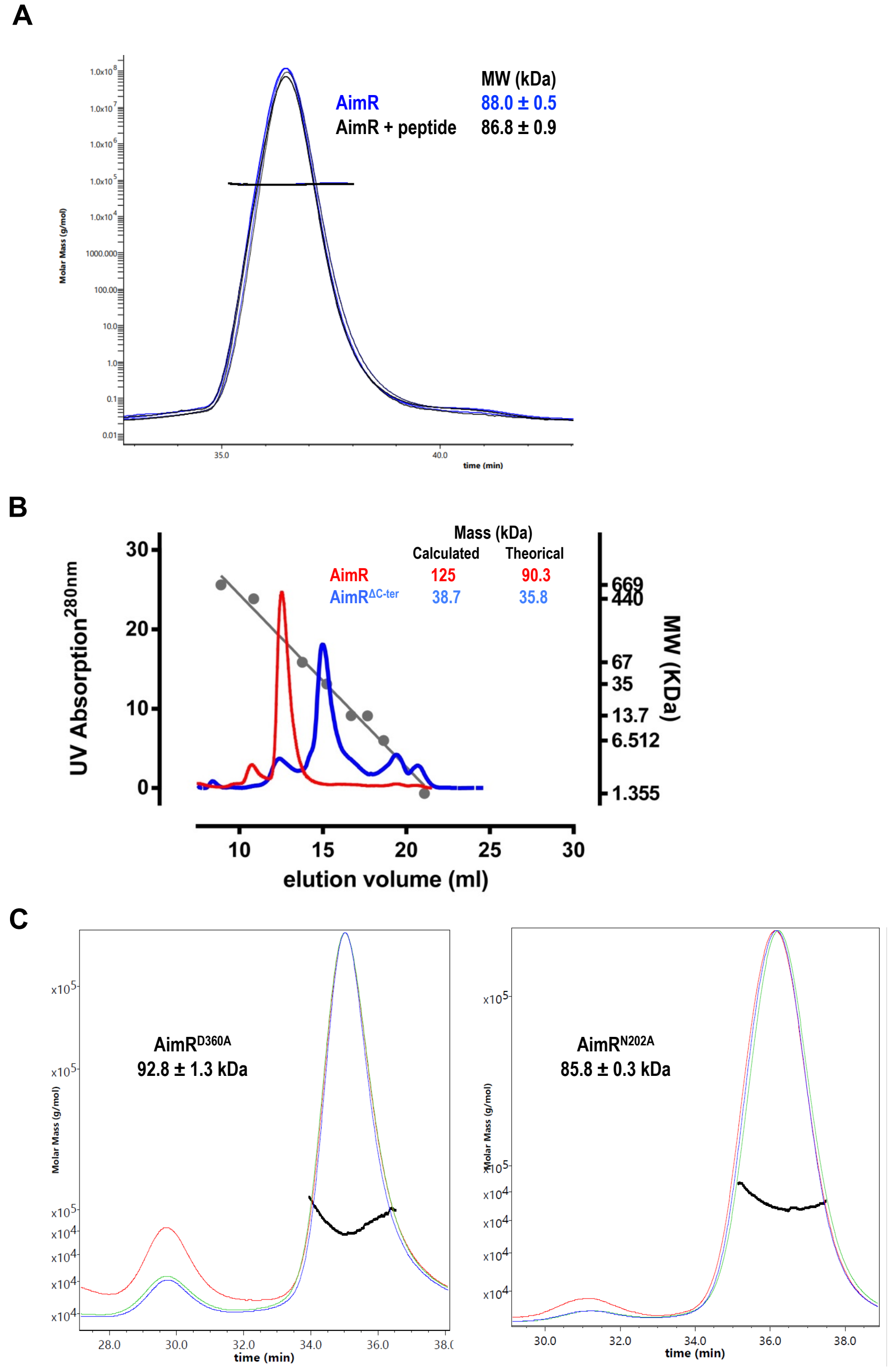

Figure S2. Size-exclusion chromatography–multi-angle light scattering (SEC-MALS) analysis of AimR. Related to Figure1. A) SEC-MALS chromatograms of AimR in absence (blue) and presence (black) of the arbitrium peptide. Chromatograms show the readings from the light scattering (dashed line), refractive index (continuous line), and UV (dotted line) detectors. The vertical axis represents the molecular mass. The horizontal curves represent the calculated molecular masses. B) Size exclusion chromatography of AimR WT and AimR$^{\Delta C\text{-ter}}$. Elution profiles of the proteins were monitored as UV absorption (red and blue curves) and the elution positions of molecular mass standards (gray circles) are plotted semilogarithmically. The molecular weight was deduced from the elution volumes (inset) and supports a dimeric organization for AimR WT and monomeric for AimR$^{\Delta C\text{-ter}}$. C) SEC-MALS chromatograms for AimR mutants N202 and D360. Chromatograms show the readings from the light scattering (greenline), refractive index (blue line), and UV (blue line) detectors.

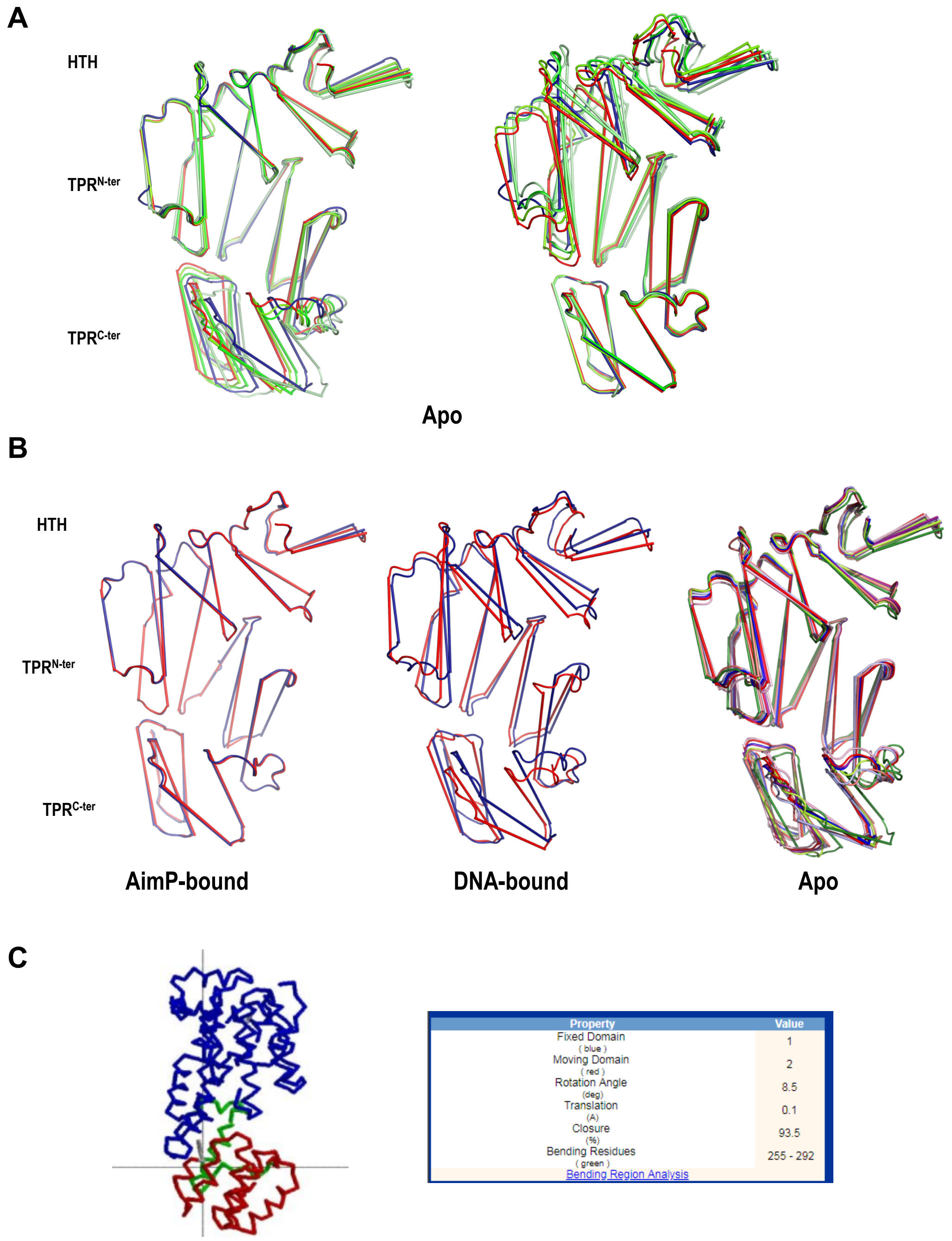

Figure S3. AimR plasticity. Related to Figures 1, 3, 4 and 6. Superimposition of the independent monomers in the different AimR structures reveals the plasticity of the protein. A) The superimposition of the (*left*) N-terminal (residues 1-263) and (*right*) C-terminal (residues 294-386) regions of the eight monomers from the AimR structure in the apo state shows an almost rigid body displacement between both regions. The structures are represented in cartoon with helices as thin cylinders. Monomers corresponding to the same dimer are colored with a different tone of the same color. B) The peptide fix the AimR structure. Superimposition of both monomers composing the dimer of AimR bound to (*left*) AimP, (*middle*) DNA or apo (*right*) shows that the peptide induces a fix conformation in both monomers (colored blue and red) while in the DNA-bound (colored blue and red) or apo (colored in tones of green, blue, red or magenta) structures each monomer present more flexibility reflected by slightly different conformations. C) DynDom analysis of AimR plasticity. Representative output of the DynDom (Hayward and Berendsen, 1998) analysis for the comparison of SPbeta AimR monomers in the four dimers of the apo state.

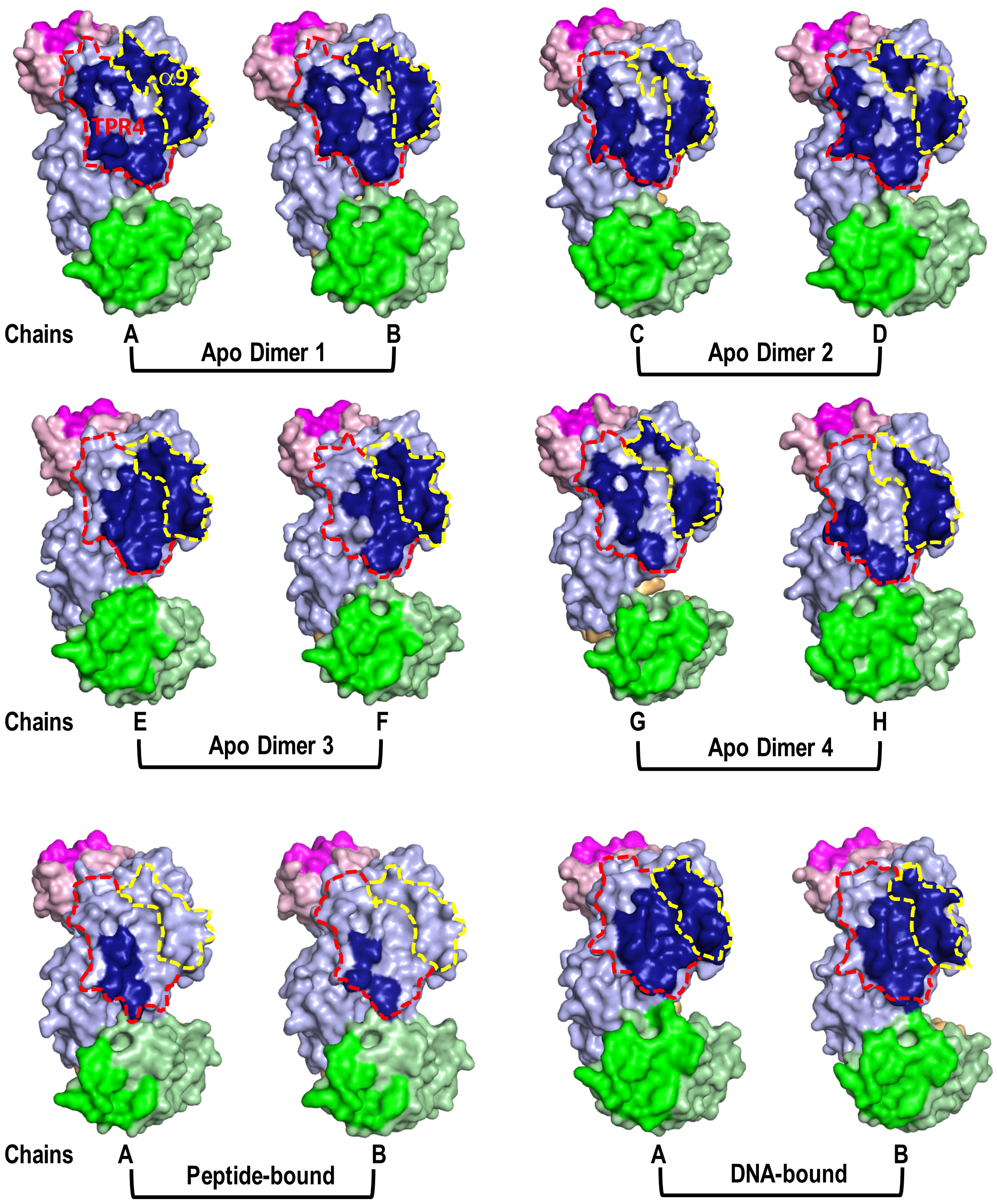

Figure S4. SPbeta AimR interface of dimerization. Related to Figures 1, 3, 4 and 6. The dimerization interface of the different AimR dimers are dissociated and individual monomers are shown in surface representation with the dimerization surface pointing towards the reader. The HTH domain, TPR$^{N\text{-}ter}$ and TPR$^{C\text{-}ter}$ subdomains and the linker are colored in pink, blue, green and orange, respectively. The dark blue-colored surfaces mark the dimerization interface provided by the TPR$^{N\text{-}ter}$ subdomain delimiting with dotted red and green lines the areas corresponding to TPR4 and α9, respectively. The bright green-colors correspond to those surfaces provided by the TPR$^{C\text{-}ter}$ subdomain

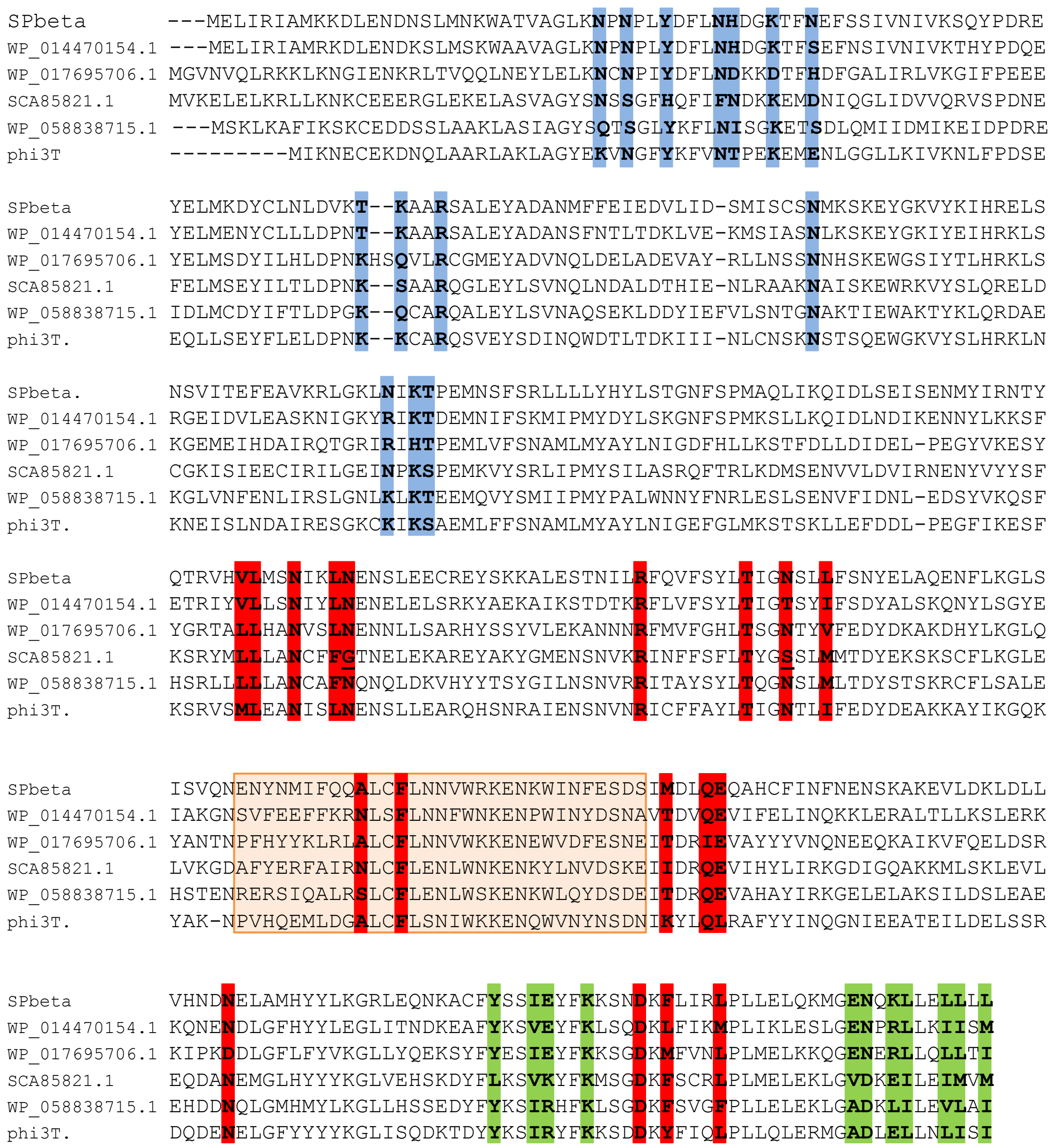

Figure S5. Sequence alignment of AimR receptors. Related to Figures 1, 3 and 4. Sequences of representatives of the six most abundant arbitrium peptide families (SPbeta for GMPRGA peptide, WP_014470154.1 for GVVRGA peptide, WP_017695706.1 for SASRGA peptide, SCA85821.1 for GFTVGA peptide, WP_058838715.1 for GFGRGA peptide and phi3T for SAIRGA peptide) were selected and aligned with Clustal Omega W (Li et al., 2015). Residues interacting with the DNA and AimP are highlighted with blue and red backgrounds, respectively. Residues from the TPR$^{C\text{-}ter}$ subdomain that are involved in dimer stabilization are highlighted with green background. The sequences corresponding to the linker region are surrounded by an orange box with background in light orange.

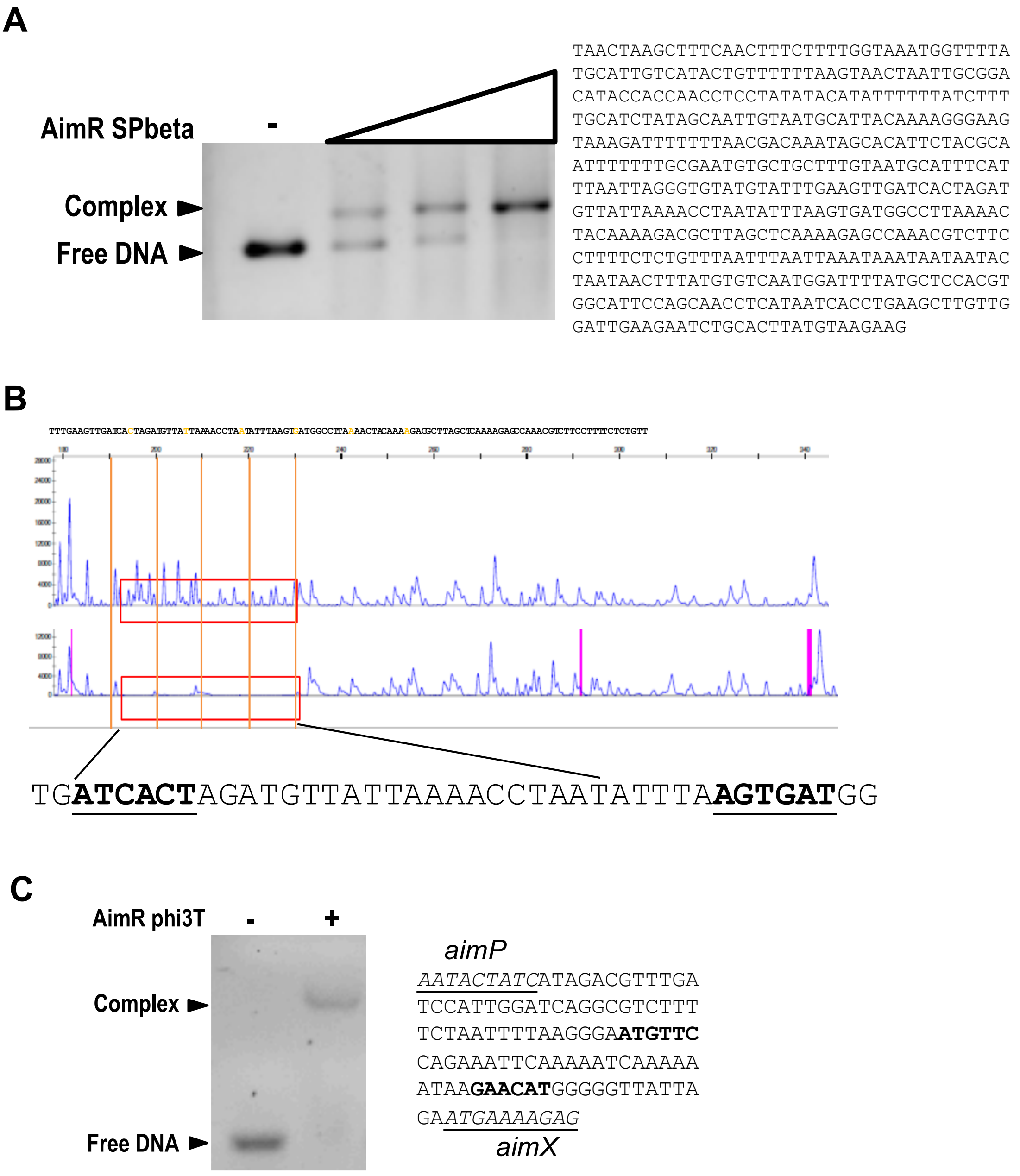

Figure S6. Characterization of SPbeta AimR operator. Related to Figure 3. A) The 359 bp DNA sequence downstream of the yopL gene (*rigth*) was proposed as DNA binding site of SPbeta AimR. EMSA assays (left) with increased amount 62.5, 125 and 250ng) of SPbeta AimR confirmed the interaction. B) DNase I footprintig analysis**.** The 359 bp region was fluorescein-labelled DNA and subjected to DNase I digestion in the presence (lower panel) or absence (upper panel) of SPbeta AimR. Fragments were analyses by capillary electrophoresis. The protected region is indicated by a red box and the DNA sequence is shown with the palindromic sequences highlighted in bold and underlined. C) The DNA sequence compressed between the *aimP* and *aimX* genes of phi3T (*left)* presents two 6 bp inverted repeated (bold letters) separated by 25 bp that is recognized by phi3T AimR (*right).*

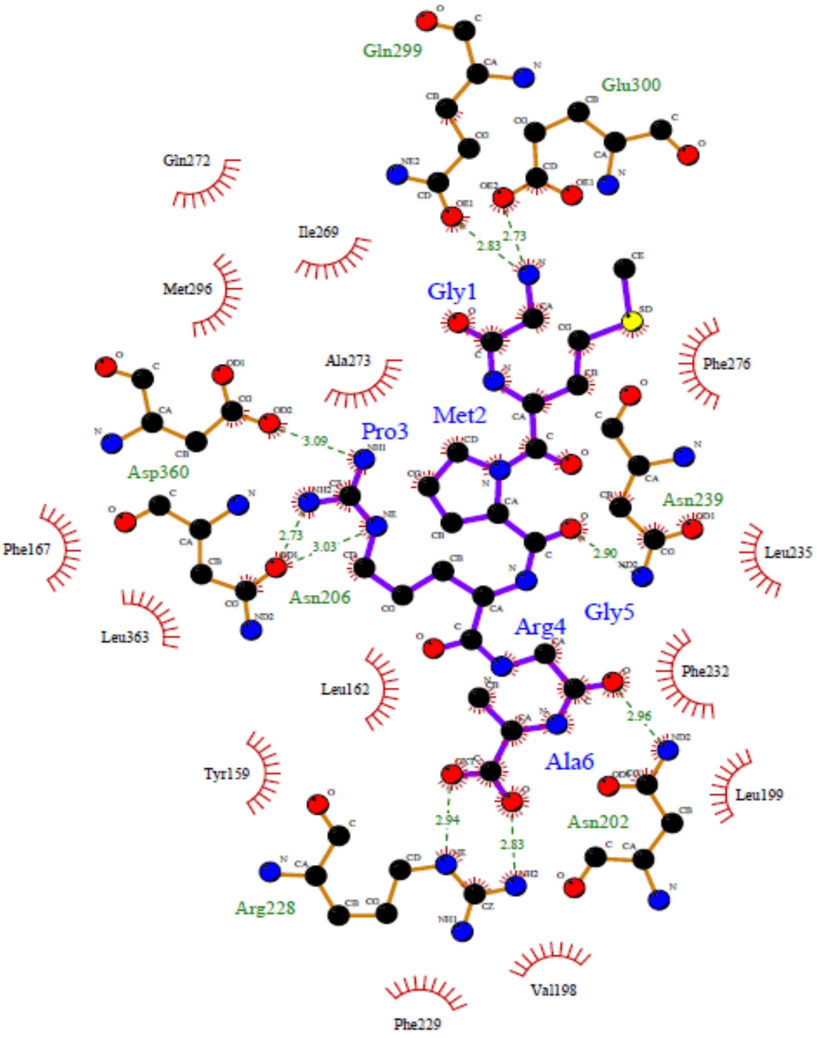

Figure S7. AimR interactions with AimP. **Related to Figure 5.** Ligplot representation of SPbeta AimR interactions with the AimP peptide. AimP bonds are shown in blue and residues are also labeled in blue. AimR atoms are connected by orange sticks and labelled in black. Polar interactions are depicted with dashed lines and bonding distances are shown.

Table S1. Oligonucleotide designs used in this study. Related to Figures 1, 3, 5 and 7, and STAR Methods.

**Oligo**	**Sequence**
**AimR cloning in pLicSGC1 plasmid**	
PlicAimR+	TACTTCCAATCCATGGAGTTAATAAGGATAGC
PlicAimR-	TATCCACCTTTACTGTTAAAGTAAAAGTAATTCTAAAAG
**AimR mutants**	
S294Stop+	TATGGATTTGCAGGAGCAAGCTC
S294Stop-	ATTTAATCAGATTCAAAATTAATCCACTTATTTTC
N202A+	TATAAAGTTAAATGAAAATTCATTAGAGG
N202A-	GCAGACATTAGAACATGAACTCTTG
D360A+	CAAATTCCTTATTAGGCTGCCAC
D360A-	GCATTAGACTTTTTAAAATACTCGATTGAAG
**Amplification of *yopR-yopS* intergenic region for EMSA assays**	
yopR/yopS+	GGAAGAAGTTAATAAATATTACGC
yopR/yopS-	GCTGCTGCTTTAGATTGTAATG
**Amplification of *yonX-yopA* intergenic region for EMSA assays**	
yonX/yopA+	GAGGGTCAGGGAATTTCGATG
yonX/yopA-	GGATTCAAGAGCTATATTTTCC
**Double stranded DNA probes for EMSA**	
[a]SPbeta WT	GTTG**ATCACT**AGATGTTATTAAAACCTAATATTTA**AGTGAT**GGCC
[b]Mut+1	GTTG**ATCACTG**GATGTTATTAAAACCTAATATTT**CAGTGAT**GGCC
Mut1	GTTG**ATCACC**AGATGTTATTAAAACCTAATATTTA**GGTGAT**GGCC
Mut2	GTTG**ATCATT**AGATGTTATTAAAACCTAATATTTA**AATGAT**GGCC
Mut3	GTTG**ATCGCT**AGATGTTATTAAAACCTAATATTTA**AGCGAT**GGCC
Mut4	GTTG**ATTACT**AGATGTTATTAAAACCTAATATTTA**AGTAAT**GGCC
Mut5	GTTG**ACCACT**AGATGTTATTAAAACCTAATATTTA**AGTGGT**GGCC
Mut6	GTTA**GTCACT**AGATGTTATTAAAACCTAATATTTA**AGTGAC**AGCC
Mut-1	GTT**AATCACT**AGATGTTATTAAAACCTAATATTTA**AGTGATA**GCC
[c]Sp-1	GTTA**ATCACT**AGATGTTATTAAAACCTAATATTT**AGTGAT**AAGCC

Sp-2	GTTAA**ATCACT**GATGTTATTAAAACCTAATATTT**AGTGAT**AAGCC
Sp-3	GTTAA**ATCACT**GATGTTATTAAAACCTAATATT**AGTGAT**TAAGCC
Sp-4	GTTAAG**ATCACT**ATGTTATTAAAACCTAATATT**AGTGAT**TAAGCC
Sp-5	GTTAAG**ATCACT**ATGTTATTAAAACCTAATAT**AGTGAT**TTAAGCC
Sp-6	GTTAAGA**ATCACT**TGTTATTAAAACCTAATAT**AGTGAT**TTAAGCC
Sp-7	GTTAAGA**ATCACT**TGTTATTAAAACCTAATA**AGTGAT**TTTAAGCC
Sp-8	GTTAAGAT**ATCACT**GTTATTAAAACCTAATA**AGTGAT**TTTAAGCC
Sp-9	GTTAAGAT**ATCACT**GTTATTAAAACCTAAT**AGTGAT**ATTTAAGCC
Sp-10	GTTAAGATG**ATCACT**TTATTAAAACCTAAT**AGTGAT**ATTTAAGCC
Sp-11	GTTAAGATG**ATCACT**TTATTAAAACCTAA**AGTGAT**ATTTAAGCC
Sp-12	GTTAAGATG**ATCACT**TATTAAAACCTAA**AGTGAT**ATTTAAGCC
Sp-13	GTTAAGATG**ATCACT**TATTAAAACCTA**AGTGAT**ATTTAAGCC
Sp+1	GTTA**ATCACT**AGATGTTATTAAAACCTAATATTTAA**AGTGAT**AGCC
Sp+2	GTTA**ATCACTA**AGATGTTATTAAAACCTAATATTTA**AAGTGAT**AGCC
Sp+3	GTTA**ATCACT**AAGATGTTATTAAAACCTAATATTTAAT**AGTGAT**AGCC
Sp+4	GTTA**ATCACTTA**AGATGTTATTAAAACCTAATATTTAAT**AGTGAT**AGCC
Sp+5	GTTA**ATCACT**TAAGATGTTATTAAAACCTAATATTTAATA**AGTGAT**AGCC
Sp+6	GTTA**ATCACT**ATAAGATGTTATTAAAACCTAATATTTAATA**AGTGAT**AGCC
Sp+7	GTTA**ATCACT**ATAAGATGTTATTAAAACCTAATATTTAATAT**AGTGAT**AGCC
Sp+8	GTTA**ATCACT**TATAAGATGTTATTAAAACCTAATATTTAATAT**AGTGAT**AGCC
Sp+9	GTTA**ATCACT**TATAAGATGTTATTAAAACCTAATATTTAATATA**AGTGAT**AGCC
Sp+10	GTTA**ATCACT**ATATAAGATGTTATTAAAACCTAATATTTAATATA**AGTGAT**AGCC
Phi3T	**ATGTTC**CAGAAATTCAAAAATCAAAAAATAA**GAACAT**

[a]SPbeta AimR operator with the 6 pb inverted repeat highlighted in bold and underlined.
[b]Mut denote SPbeta AimR operators with mutations in the inverted repeats highlighted in red.
[c]Sp denote SPbeta AimR operators with spacers of variable length.

Table S2. Putative AimR operators in SPbeta genome. Related to Figures 7 and 4, and STAR Methods.

Sequence	SPbeta Genome		Gene
	from	to	
23 bp Spacer			
[a]ATCACTTAATTTAAAATTTTCAACTGATCCGTCAG	82995	83029	*yopT*
24 bp Spacer			
ATCACTTGCTGATTTAGCTTCACCACCAGCTGTTTT	35613	35648	*yomI*
25 bp Spacer			
[b]ATCACTAGATGTTATTAAAACCTAATATTTAAGTGAT	**77707**	**77743**	*yopL-yopM*
ATCACTTATCTCCTTTCATCGTTCACAAGCATAGTTGT	5567	5604	*yofK*
ATCACTTACTCTCCATCATCATCATCTGTAACAGTATC	119220	119258	*yosH*
ATCACTTGGTCAATAAACCCGTACTGAAGGGCAGTTAA	58271	58308	*yonJ*
26 bp Spacer			
ATCACTTACTTGGTGTTTGCTGCCCCTTTGCAAGTGTG	73127	73163	*yopD*
ATCACTAACAAGTAAATAGTTATTTCTAATTTCGTATA	17287	17324	*yolH*
ATCACTTAGTAAATCAGCTAAGACCTGATCCATGTCAA	113016	113053	*yorS*
27 bp Spacer			
ATCACTCTCCTAATCTATATTGAAGGTAAATTATGTATG	133553	133591	*yotL-yotM*
28 bp Spacer			
[c]ATCACTTTGTTAAAGCTAATTCCTATCCTTCCCAGGTAAT	69299	69337	*yonX-yopA*
ATCACTAATCGTTATAAGCCCCTGTAATTAATTTTGTAGT	120440	120479	*yosL*
ATCACTCATCTTGAGTGCCTCCTTAGATAAACAATGTGCT	118316	118355	*yosE-yosF*
ATCACTGTGTGTTATTACATTTCTGTTTTTATCACGTTTA	28823	28862	*yomG*
[c]ATCACTTTAAATACATGTTTAAATGTAGTAAGATGGTCAT	82354	82393	*yopR-yopS*
ATCACTTTAATATTCATAAGTATGGTCTTAATAAGGTGCA	17607	17646	*yolH*

[a]Inverted repeats are underlined and the nucleotide conserved with AimR operator are highlighted in bold red letters.
[b]SPbeta AimR operator
[c]Alternative operators tested by EMSA
